# Comparing the Effectiveness of Different Dietary Educational Approaches for Carbohydrate Counting on Glycemic Control in Adults with Type 1 Diabetes: Findings from the DIET-CARB Study, a Randomized Controlled Trial

**DOI:** 10.3390/nu16213745

**Published:** 2024-10-31

**Authors:** Bettina Ewers, Martin Bæk Blond, Jens Meldgaard Bruun, Tina Vilsbøll

**Affiliations:** 1 Department of Diabetes Care, Copenhagen University Hospital, Steno Diabetes Center Copenhagen, DK-2730 Herlev, Denmark; tina.vilsboell.01@regionh.dk; 2 Department of Clinical & Translational Research, Copenhagen University Hospital, Steno Diabetes Center Copenhagen, DK-2730 Herlev, Denmark; martin.baek.blond@regionh.dk; 3 Steno Diabetes Center Aarhus, Aarhus University Hospital, DK-8200 Aarhus, Denmark; jens.bruun@clin.au.dk; 4 Department of Clinical Medicine, University of Aarhus, DK-8200 Aarhus, Denmark; 5 Faculty of Health and Medical Sciences, University of Copenhagen, DK-2200 Copenhagen, Denmark

**Keywords:** carbohydrates, carbohydrate counting, glycemic control, group-based education, HbA1c, individual dietary counselling, MAGEs, patient education, type 1 diabetes

## Abstract

Background/Objectives: Carbohydrate counting is recommended to improve glycemic control in type 1 diabetes (T1D), but the most effective educational methods are unclear. Despite its benefits, many individuals struggle with mastering carbohydrate counting, leading to inconsistent use and suboptimal glycemic outcomes. This study aimed to compare the effectiveness of two group-based programs with individual dietary counseling (standard care) for glycemic control. Methods: The trial was a randomized, controlled, open-label, parallel-group design. Adults with T1D on multiple daily insulin injections (MDIs) and with glycated hemoglobin A1c (HbA1c) 53–97 mmol/mol were randomly assigned (1:1:1) to basic (BCC), advanced carbohydrate counting (ACC), or standard care. Primary outcomes were the changes in HbA1c or mean amplitude of glycemic excursions (MAGEs) in BCC and ACC versus standard care after six months. Equivalence testing was performed to compare BCC and ACC. Results: Between November 2018 and August 2021, 63 participants were randomly assigned to BCC (N = 20), ACC (N = 21), or standard care (N = 22). After 6 months, HbA1c changed by −2 mmol/mol (95% CI −5 to 0 [−0.2%, −0.5 to 0]) in BCC, −4 mmol/mol (−6 to −1 [−0.4%, −0.6 to −0.1]) in ACC, and −3 mmol/mol (−6 to 0 [−0.3%, −0.6 to 0]) in standard care. The estimated difference in HbA1c compared to standard care was 1 mmol/mol (−3 to 5 [0.1%, −0.3 to 0.5]); *p* = 0.663 for BCC and −1 mmol/mol (−4 to 3 [−0.1%, −0.4 to 0.3]); *p* = 0.779 for ACC. For MAGEs, changes were −0.3 mmol/L (−1.5 to 0.8) in BCC, −0.0 mmol/L (−1.2 to 1.1) in ACC, and −0.7 mmol/L (−1.8 to 0.4) in standard care, with differences of 0.4 mmol/L (−1.1 to 1.9); *p* = 0.590 for BCC and 0.7 mmol/L (−0.8 to 2.1); *p* = 0.360 for ACC versus standard care. An equivalence in effect between BCC and ACC was found for HbA1c, but not for MAGEs. Conclusions: Group-based education in BCC and ACC did not demonstrate a clear advantage over individualized dietary counseling for overall glycemic control in adults with T1D. Healthcare providers should consider flexible, patient-centered strategies that allow individuals to choose the format that best suits their learning preferences when selecting the most suitable dietary educational approach.

## 1. Introduction

Effective treatment of individuals with type 1 diabetes (T1D) relies on continuous self-management and evidence-based nutrition education tailored to individual needs [1]. This nutrition education aims to empower individuals by providing them with essential skills, knowledge, confidence, and autonomy to manage the complexities of T1D, particularly in relation to dietary intake, blood glucose levels, and prandial insulin dosing [1]. Carbohydrate counting stands out as a key aspect of diabetes-specific nutrition education for individuals with T1D on multiple daily injections (MDIs) insulin therapy, as recommended by international guidelines [2,3,4]. It involves meticulously tracking and counting the carbohydrate intake in grams throughout the day, enabling individuals to more effectively adjust their mealtime insulin doses and regulate their blood glucose levels. Despite its importance, uncertainties persist regarding the impact of different educational approaches to carbohydrate counting, particularly concerning the most effective delivery methods and influence on clinical outcomes. Two distinct levels of carbohydrate counting, basic (BCC) and advanced (ACC), have been defined, each highlighting different approaches and levels of complexity [5]. BCC focuses on maintaining a consistent carbohydrate intake in terms of type, amount, and distribution throughout the day, using a more intuitive and flexible approach to mealtime insulin adjustments based on prescribed insulin doses. In contrast, ACC involves personalized mealtime insulin adjustments based on carbohydrate intake using algorithms. Ideally, effective carbohydrate counting management requires accurate calculation and subsequent mealtime insulin dosing based on carbohydrate-to-insulin ratios, insulin sensitivity, and other factors (e.g., physical activity), encompassing aspects of both BCC and ACC. However, barriers such as a lack of motivation, low numeracy or literacy, and inaccurate carbohydrate estimations have been associated with poorer glycemic control using ACC [6,7,8,9,10,11]. While technologies such as bolus calculator apps for smartphones aim to simplify ACC for individuals with T1D treated with MDIs therapy, they do not fully eliminate the need for individuals to self-estimate carbohydrate portion sizes, which impact glycemic outcomes [12,13,14]. Moreover, empirical data, including clinical observations, show that ACC may be too complex for some individuals to manage effectively. Despite the proven efficacy of ACC in reducing HbA1c [15,16,17], evidence on the educational impact of BCC remains limited. This knowledge gap is particularly important, as BCC may offer a more feasible approach for some individuals, potentially leading to better adherence and improved glycemic outcomes. Additionally, while group-based dietary educational approaches show promise, they are relatively underexplored compared with individual dietary counseling, which remains the standard of dietary care worldwide [18,19]. Understanding the comparative effectiveness of BCC and ACC, particularly in group settings, may be crucial for optimizing dietary education strategies and improving glycemic control in T1D. Accordingly, our study aimed to investigate the efficacy of two dietitian-led, group-based educational approaches for carbohydrate counting (BCC and ACC) compared with individual dietary counseling (standard of dietary care) on improving glycemic control among adults with T1D treated with MDIs after six months of treatment. We hypothesized that both BCC and ACC would be superior to standard dietary care in reducing HbA1c or the mean amplitude of glycemic excursions (MAGEs). Additionally, we hypothesized that BCC would be equivalent to ACC in reducing HbA1c or MAGEs.

## 2. Materials and Methods

### 2.1. Study Design and Participants

A detailed description of the trial has been previously published in a protocol paper [20]. The study was a single-center, parallel-group, randomized, controlled, open-label, superiority trial conducted at Steno Diabetes Center Copenhagen, a tertiary health care facility in the Capital Region of Denmark, over a 12-month period. Inclusion criteria comprised individuals aged 18–75 years, diagnosed with T1D and treated in an outpatient diabetes clinic in the Capital Region of Denmark, undergoing treatment with MDIs, having a diabetes duration exceeding 12 months, and an initial HbA1c level of 53–97 mmol/mol. Exclusion criteria included currently practicing carbohydrate counting or a low daily carbohydrate intake (defined as <100 g per day), engagement in a carbohydrate counting program within the past two years, use of an automated bolus calculator or insulin pump, planning to initiate insulin pump therapy during the study period, use of split-mixed insulin therapy, gastroparesis, uncontrolled medical issues affecting dietary intake, pregnancy or lactation, planning pregnancy during the study period, involvement in other clinical trials, and inability to comprehend the informed consent or the study procedures. The screening and study visits (at baseline, after the six-month intervention, and at six-month follow-up) were conducted by the study personnel. The inclusion period spanned from October 2018 to August 2021. This study was approved by the Scientific Ethics Committee in the Capital Region of Denmark and the trial is registered at ClinicalTrials.gov (NCT03623113).

### 2.2. Screening and Randomization

Individuals interested in participating attended a screening visit. During the screening visit, eligible participants were randomly assigned in a 1:1:1 ratio by the study investigator or personnel to receive either BCC, ACC, or standard dietary care (control group) through the use of a randomization module [20]. The randomization module was based on a randomization list that was generated and uploaded to the electronic data management system REDCap (version 8.10.18, Vanderbilt University, Nashville, TN, USA) by an external statistician prior to the commencement of the trial.

### 2.3. Interventions

Participants assigned to standard dietary care attended three individual dietary counselling sessions, totaling 2 h, conducted at week 0, 2, and 12. In these sessions, personal dietary goals were established based on the overall metabolic goal, preferences for dietary adjustments were explored, guidance on carbohydrate awareness including glycemic index of foods, meal planning, and portion management was provided, and personal queries or worries regarding dietary management of T1D were addressed.

Participants in the BCC group attended three group sessions (4–8 participants), including a total duration of 8 h, held at week 0, 2 and 12. This structured group-based BCC program was designed to empower participants in managing their postprandial blood glucose levels by regulating their carbohydrate intake. The program included concise theoretical presentations on food and nutrition in relation to diabetes, engaging in problem-solving exercises, and practical sessions focusing on identifying carbohydrates and estimating carbohydrate portion sizes across various foods. Participants explored different methods of carbohydrate monitoring, including the use of nutrition labels, interpreting carbohydrate tables, and employing smartphone applications. Additionally, participants were instructed to keep a dietary log to track carbohydrate intake and blood glucose levels over a 4-day period, facilitating the development of a personal carbohydrate plan. The program also included discussions on dietary coping strategies and incorporated peer modeling and support.

Participants in the ACC group attended one group session (4–8 participants), lasting 4 h, held at week 0, followed by two individual dietary follow-up sessions totaling 1.5 h conducted at week 2 and 12. The ACC program included instruction on how to use an automated bolus calculator (MySugr Pro. Roche Diabetes, app available in Google Play and AppStore). The bolus calculator was set with personalized ratios for the insulin sensitivity factor (for blood glucose adjustments) and carbohydrate-to-insulin dosing at meals. These ratios were estimated by a dietitian based on each participant’s 7-day dietary recordings, including blood glucose measurements and mealtime insulin dosages. The teaching approach integrated theoretical and practical training, drawing on real-life examples and experiences with T1D.

Sessions in all three study groups were conducted by the same trained dietitians, following a structured curriculum and supervision by an endocrinologist if necessary. Further details regarding the BCC and ACC programs, as well as standard dietary care, are available in the protocol paper [20]. Participants were advised to maintain consistent physical activity patterns throughout the study, while medical adjustments, including insulin adjustments, were permitted when needed.

### 2.4. Compliance

Participants in the ACC group were advised to use the automated bolus calculator for meals with 15 g or more of carbohydrates, while participants in the BCC group were instructed to follow their personal carbohydrate plan daily for all meals [20]. Compliance with automated bolus calculator usage in the ACC group was assessed through exported app data indicating each instance usage. Compliance with the personal carbohydrate plan in the BCC group was assessed based on the question: “How often do your meals deviate from your personal carbohydrate plan prescribed by the dietitian?”, using a visual analogue scale (VAS) ranging from never (0) to always (100). Compliance was not assessed for the standard dietary care group.

### 2.5. Outcome Measures

The primary outcomes included changes in HbA1c and MAGEs, with the latter measuring glycemic variability, from baseline to end-of-treatment at six months. Secondary and exploratory outcomes included changes from baseline to end-of-treatment at six months and after six months of follow-up in other clinically relevant metabolic markers including time in range (TIR), time below range (TBR), time above range (TAR), coefficient of variation (CV), mean plasma glucose based on data from a blinded continuous glucose monitoring (CGM) device, and HbA1c (at six months follow-up), as well as body weight, body composition, blood pressure and lipid profile, total insulin dose, prandial insulin dose, and basal insulin dose.

Additionally, changes in skills related to numeracy and carbohydrate estimation accuracy, patient-reported outcomes (diabetes diet-related quality of life (DDQOL), perceived dietitian-related autonomy support (HCCQ), and competencies in diet and diabetes (PCDS)) and behavioral outcomes (dietary changes in intake of total energy, macronutrients, added sugar, and dietary fibers based on 4-day dietary recordings), and changes in level of physical activity assessed by the Danish version of the International Physical Activity Questionnaire—Short Form (IPAQ-SF) [21] were assessed from baseline to end-of-treatment at six months and after six months of follow-up (diet only at baseline and after six months intervention). Details regarding these outcomes can be found in the protocol paper [20].

### 2.6. Sample Size

The trial was designed with 80% statistical power (α = 0.05) to detect a difference in HbA1c of 3.5 mmol/mol (SD 7 mmol/mol) between the BCC group and the standard care group or the ACC group and the standard care group. This determination was primarily informed by findings from experimental studies investigating the impact of BCC [22,23,24] and meta-analyses of RCTs evaluating the effect of ACC [15,16] compared with a control or usual dietary care group. These studies found HbA1c reductions ranging from 3 to 7 mmol/mol in adults with T1D. Notably, participants in these studies exhibited poorer diabetes control (60−108 mmol/mol) compared with our study’s eligible participants (53–97 mmol/mol). Thus, we anticipated smaller HbA1c reductions in our study population but still deemed them clinically significant within a multidisciplinary approach for managing hyperglycemia in T1D. The clinical target for MAGEs in T1D is still unknown [25], but the trial was designed to detect a difference of ≥0.35 mmol/L (SD 0.7 mmol/L) in MAGEs between the groups [26]. Taking these assumptions into account, along with an anticipated 20% dropout rate, the required sample size was calculated to include 231 participants in total, with 77 participants assigned to each group.

### 2.7. Changes Due to the COVID-19 Pandemic

Due to the COVID-19 pandemic, all non-urgent outpatient appointments were transitioned to virtual appointments from March until September 2020, and again from November 2020 to February 2021, due to a resurgence in COVID-19 cases. Additionally, scheduled study visits for enrolled participants were postponed during these lockdown periods. Consequently, most visits, particularly the final six-month follow-up visits, were delayed and spread out over a longer period than originally planned. As a result, the trial stopped participant recruitment in September 2021 before reaching the intended sample size and without prior data review [27].

### 2.8. Statistical Analyses

Baseline data are reported as means with standard deviations (SD) for continuous variables following a normal distribution and as medians with interquartile ranges (25th and 75th percentiles) for non-normally distributed variables. Categorical variables are presented as frequencies and percentages. Intention-to-treat analyses, using all available data, were performed to compare treatment effects across the study groups for the prespecified primary outcomes, HbA1c and MAGEs, and selected secondary and exploratory outcomes. Treatment effects are presented as baseline-adjusted differences between groups for all outcomes. Linear mixed-effects models were used to model the outcomes, with baseline corrections made by setting all participants in the control group at baseline. Fixed effects included visit and the interaction between treatment group and visit. Before estimating treatment effects, residuals were evaluated graphically to check assumptions of normality and homogeneity of variances. Where needed, outcomes were log-transformed for analysis and subsequently back-transformed for presentation. The estimated mean differences in changes (with 95% confidence intervals) between and within groups are provided, along with two-sided p-values. Equivalence testing was conducted to compare the effect of BCC versus ACC on HbA1c and MAGEs. Equivalence was established if the 90% confidence intervals (CI) for the estimated difference in chance in HbA1c or MAGEs between the two groups fell entirely within the predefined equivalence margins according to the statistical analyses plan (Supplementary Materials File S1). If the confidence interval exceeded these margins in either direction (negative or positive), equivalence was not claimed. The analysis was performed using the same linear mixed-effects model described above. Non-parametric tests (Wilcoxon) were used to assess changes in summed scores from baseline to the end of treatment for the three psychometric tests (DDQOL, HCCQ, and PCDS) due to the non-normal distribution of the data. Statistical significance was determined using a two-tailed *p*-value of <0.05. The false discovery rate (FDR) for secondary and exploratory outcomes was controlled with the Benjamini and Hochberg method, applying a threshold of <5% [28]. Missing data were handled using maximum likelihood estimation in the linear mixed model, under the assumption that the data were missing at random. All statistical analyses were conducted using SAS Enterprise Guide version 8.3 Update 3 (SAS Institute Inc., Cary, NC, USA) and R software version 4.0.2 (R Core Team, R Foundation for Statistical Computing, Vienna, Austria).

## 3. Results

We assessed 144 individuals for eligibility: 48 either declined the invitation or did not respond and 33 did not meet the inclusion criteria. Ultimately, 63 participants were enrolled and randomly assigned: 20 to the BCC group, 21 to the ACC group, and 22 to the standard care group (control). A flow diagram, included as supplementary material (Figure S1), illustrates the number of participants who dropped out or were lost to follow-up in each group during the various phases of the trial, along with the reasons for these occurrences. Analyses are based on data from 53 out of the 63 participants, as 10 participants (BCC, *n* = 2; ACC, *n* = 3; standard care, *n* = 5) withdrew before baseline data had been collected. Table S1 displays the clinical and sociodemographic data for individuals who dropped out before baseline measurements. Data were collected during the screening visit after study inclusion. In general, more women and participants with a higher HbA1c dropped out. The baseline characteristics of the participants by allocation are shown in Table 1 and Supplementary Materials in Table S2. Overall, the study population had an average age around 44 years with 70% males, an average diabetes duration of 17 years, and moderately uncontrolled glycemic regulation with a median HbA1c of 64 mmol/mol. Fifty-five percent used a CGM or a flash glucose monitoring (FGM) device at inclusion. During the intervention period, seven participants initiated the use of GCM/FGM (n = 3 in ACC, n = 2 in BCC, and n = 2 in standard care). At follow-up, one more participant in ACC, one more in BCC, and two more in standard care had initiated CGM/FGM use.

Three participants had been prescribed glucagon-like peptide 1 receptor agonists (GLP−1RAs), while 25% were on antihypertensive medication, and 32% had been prescribed lipid-lowering medication (Table 1). Few changes in these prescribed drugs were observed during the study period (Table S3). The average number of outpatient diabetes clinic visits with an endocrinologist during the intervention period was 1.3 for the BCC group, 1.1 for the ACC group, and 1.0 for the standard care group. For nurse consultations, the averages were 1.2 (BCC), 0.9 (ACC), and 0.8 (standard care). Additionally, participants attended an average of 2.9 group-course dietitian sessions (BCC), 3.1 mixed group and individual sessions (ACC), or 3.0 individual dietitian consultations (standard care). During the follow-up period, the average number of visits with an endocrinologist was 0.9 for the BCC group, 1.1 for the ACC group, and 0.8 for the standard care group. Nurse consultations averaged 0.5 (BCC), 0.9 (ACC), and 0.8 (standard care), while dietitian visits were 0.0 (BCC), 0.1 (ACC), and 0.1 (standard care). Table S4 presents self-reported data on the frequency and methods used (experience-based versus carbohydrate calculations) for mealtime insulin dosing at baseline, end-of-treatment, and follow-up for all study groups.

### 3.1. Compliance

Data from the automated bolus calculator indicated that 60% of participants in the ACC group utilized the bolus calculator app multiple times daily, 13% used it several days per week, and the remaining 27% employed the app more intermittently during the intervention period. Between end-of-treatment and follow-up, 50% of participants still used the bolus calculator multiple times daily, 14% used it intermittently, and 36% had stopped using the bolus calculator app. In the BCC group, participants reported using the personal carbohydrate plan 51% of the time (IQR 24, 73) during the intervention period and 48% of the time (IQR 38, 61) at follow-up.

### 3.2. Primary Outcomes

**Figure 1:**
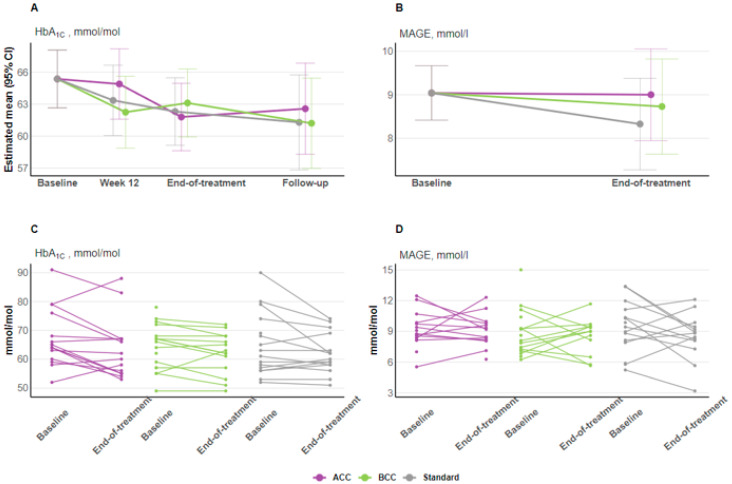
Primary outcomes. (**A**) Changes in estimated means (CI 95%) for HbA1c from baseline to end of follow-up for all groups; (**B**) Changes in estimated means (CI 95%) for MAGEs from baseline to end of follow-up for all groups; (**C**) Spaghetti plots showing individual HbA1c changes from baseline to end-of-treatment for completers across all groups; (**D**) Spaghetti plots showing individual MAGEs changes from baseline to end-of-treatment for completers across all groups. Abbreviations: ACC, advanced carbohydrate counting; BCC, basic carbohydrate counting; HbA1c, hemoglobin A1c; MAGEs, mean amplitude of glycemic excursions.

Compared with standard care, no treatment effects were observed for the BCC intervention on HbA1c (1 mmol/mol (−3 to 5 [0.1%, −0.3 to 0.5]); *p* = 0.663), or MAGEs (0.4 mmol/L (−1.1 to 1.9); *p* = 0.590), nor for the ACC intervention on HbA1c (−1 mmol/mol (−4 to 3 [−0.1%, −0.4 to 0.3]); *p* = 0.779) or MAGEs (0.7 mmol/L (−0.8 to 2.1); *p* = 0.360) from baseline to end-of-treatment at six months (Figure 1A,B, Table 2). Individual changes in HbA1c and MAGEs from baseline to end-of-treatment for completers in all three study groups are shown in Figure 1C,D.

For HbA1c, the 90% CI for the estimated difference in chance between BCC and ACC was −1.23 to 1.78 mmol/mol. This did not surpass the predefined equivalence margin of ±3.5 mmol/mol. For MAGEs, the 90% CI for the estimated difference in chance between BCC and ACC was −5.02 to 2.38 mmol/L, which surpass the predefined equivalence margins of ±0.35 mmol/L. These results indicate an equivalence in effect between the BCC and ACC interventions on HbA1c, but not on MAGEs, suggesting the two interventions may differ in their effect on this outcome.

### 3.3. Secondary and Exploratory Outcomes

**Figure 2:**
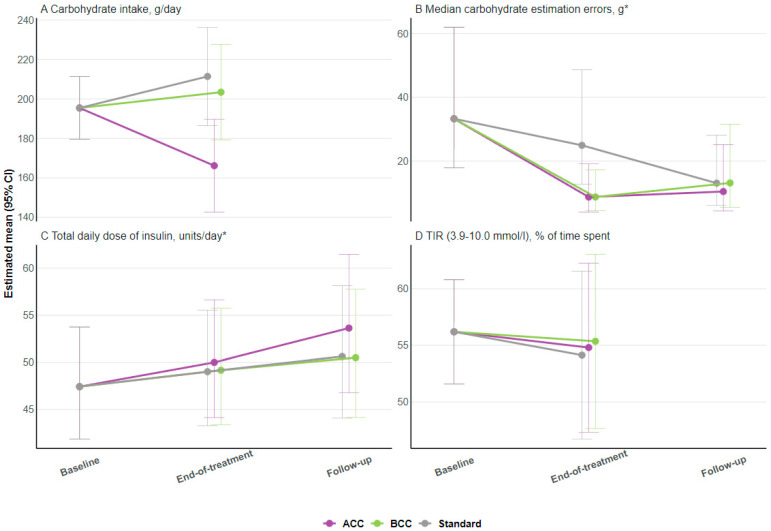
Changes in estimated means (CI 95%) for selected secondary outcomes. (**A**) Carbohydrate intake, g/day; (**B**) median carbohydrate estimation errors, gram; (**C**) total daily dose of insulin, units/day; and (**D**) TIR (3.9−10.0 mmol/L), % of time spent. Abbreviations: ACC, advanced carbohydrate counting; BCC, basic carbohydrate counting; TIR, time in range. * Log-transformed for analysis and back-transformed for presentation.

**Figure 3:**
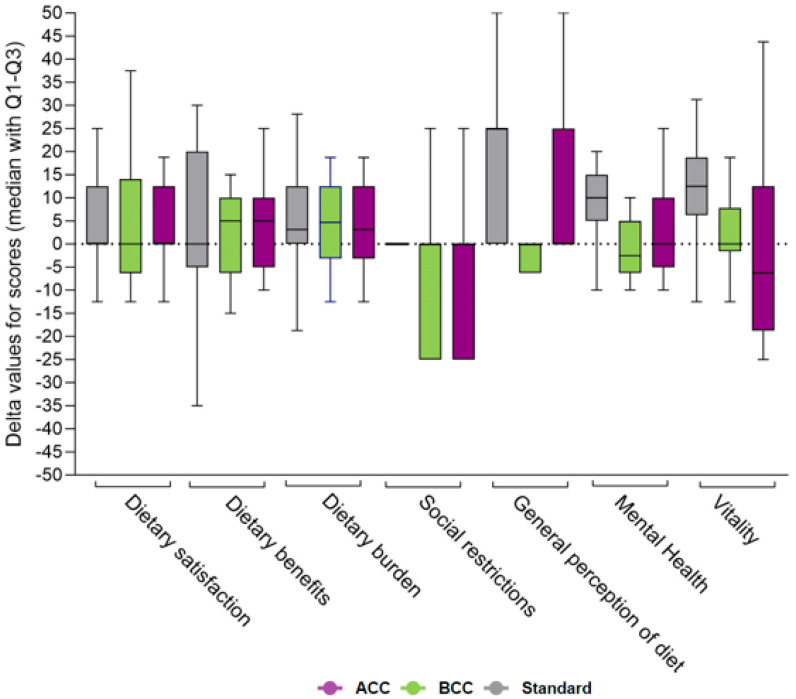
Diabetes diet –related quality of life (DDQOL) presented as delta values (median with Q1 and Q3) for summed scores from baseline to end of treatment across the seven subdomains. Abbreviations: ACC, advanced carbohydrate counting; BCC, basic carbohydrate counting.

**Figure 4:**
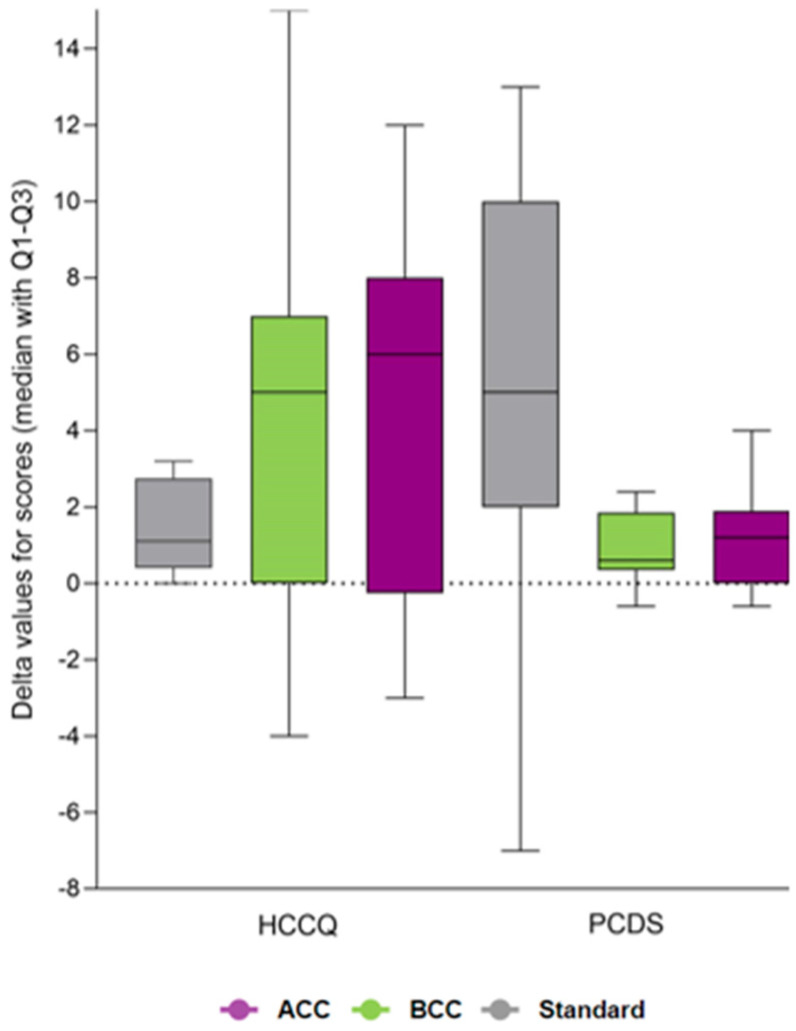
Health Care Climate Questionnaire (HCCQ) and Perceived Competencies in Diet and Diabetes Scale (PCDS) presented with delta values (median with Q1 and Q3) for summed scores from baseline to end of treatment. Abbreviations: ACC, advanced carbohydrate counting; BCC, basic carbohydrate counting.

No differences in secondary or exploratory outcomes were found between the study groups, except for total energy intake and saturated fat intake, which remained significant in favor of ACC after multiple testing adjustments. The estimated treatment difference was −10 g/day (95% CI: −16 to −5; *p* < 0.001) for saturated fat and −2204 kJ/day (95% CI: −3281 to −1126; *p* < 0.001) for total energy intake (shown in Table S5). Changes from baseline to end-of-intervention in carbohydrate intake, median carbohydrate estimation errors, insulin dose, and time-in-range are presented in Figure 2A,D. Delta values for changes in person-reported outcomes from baseline to end-of-intervention are presented in Figure 3 for diabetes diet-related quality of life (DDQOL) and Figure 4 for perceived dietitian-related autonomy support (HCCQ) and competencies in diet and diabetes (PCDS). Additional supplementary secondary/exploratory outcomes are presented in Tables S5 and S6.

## 4. Discussion

Our study found that group-based education in ACC and BCC did not lead to improvements in glycemic control, as measured by HbA1c and MAGEs, when compared with individual dietary counseling for individuals with T1D treated with MDIs. ACC and BCC differed in their effect on MAGEs, but not in their effect on HbA1c. No further relevant effects were seen for the secondary or exploratory outcomes. These findings contrast with systematic reviews and meta-analyses based on up to six randomized trials, which have reported HbA1c reductions between 4 mmol/mol (0.4%) to 7 mmol/mol (0.6%) in favor of ACC when compared with a control group in adults with T1D, although substantial heterogeneity was reported across the included trials [15,16,29]. The fact that the estimated target sample size was not reached in our study may have contributed to the lack of observed effects, leading to inconclusive study results. However, key differences in study design and the patient populations may have influenced the results. Notably, previous randomized trials often included participants with more poorly controlled T1D, characterized by higher baseline HbA1c levels (≥59 mmol/mol (7.5%)), as an inclusion criterion [13,30] and used general diabetes education or usual care as the control, without any dietary intervention [13,23,30,31,32]. In contrast, our study used individual dietary counseling as the control. These differences may explain some of the observed variation in outcomes, suggesting that group-based carbohydrate counting interventions may be less effective for individuals with moderately uncontrolled T1D.

Interestingly, all three study groups showed improvements in glycemic regulation during both the intervention and follow-up periods. While this may reflect a study or time effect, it is also possible that the uniform improvement across all study groups suggests that the dietary interventions, regardless of their format, had a beneficial impact on glycemic regulation.

No changes in the ability to estimate carbohydrates accurately were found after both BCC and ACC interventions compared with standard dietary care. In our previous study involving participants with uncontrolled type 2 diabetes, we found that participants significantly improved their carbohydrate estimation skills after BCC education; however, their baseline skills were notably poorer compared with our participants with T1D [33]. This aligns with the observation that most individuals with T1D have, at some point, engaged in improving their carbohydrate assessment skills after diagnosis, whereas this has not traditionally been a focus in type 2 diabetes management.

We found that total energy intake and intake of saturated fat were reduced in the ACC group compared with the standard dietary care group, even after adjusting for multiple testing. However, these reductions did not translate into clinically meaningful improvements, such as greater weight loss associated with lower energy intake or reductions in plasma cholesterol levels following the reduction in saturated fat intake in the ACC group at the end of the intervention. These changes in dietary outcomes may be influenced by the inherent limitations of self-reported dietary intake, which is often subject to inaccuracies, particularly underreporting and selective misreporting (e.g., the underreporting of unhealthy foods high in sugar and fat and the overreporting of healthy foods such as vegetables) [34,35]. These inaccuracies also complicate the interpretation of the effects of dietary changes on clinical outcomes.

This study faced several important limitations, including a substantially smaller sample size than planned and delays in study visits due to COVID-19, both of which reduced the statistical power of our trial. The observed differences in the effects of BCC and ACC on MAGEs may also be attributed to the lack of power, as the width of the confidence intervals heavily influenced by sample size. Additionally, there was a high dropout rate after study inclusion but prior to study commencement, particularly in the standard dietary care group, which may have biased the results. This dropout does not seem to be primarily due to a preference for group interventions, as only one participant explicitly cited this reason. Instead, most dropouts across all groups were attributed to external factors such as COVID-19 and personal issues. This suggests that the mode of intervention (individual vs. group) was likely not an important factor in participant retention, with external circumstances playing a more prominent role.

The BCC program was designed to improve carbohydrate counting accuracy and ensure day-to-day consistency of carbohydrate intake according to a personalized carbohydrate plan, reducing carbohydrate overload at each meal. Meanwhile, the ACC program aimed to enhance mealtime insulin dose accuracy through the use of an automated bolus calculator. However, compliance data indicated that only approximately 60% of participants in the ACC group used their bolus calculator app several times daily during the study. Similarly, self-reported data showed that use of the personal carbohydrate plan was moderate in the BCC group, suggesting limited use by a notable proportion of participants. This lack of sustained adherence to the dietary interventions may be attributed to issues of acceptability, feasibility, and insufficient motivation to fully engage with the prescribed methods. Considering that participants in all three study groups had only three visits with a dietitian, either in groups or individually, ongoing support and follow-up, such as regular check-ins via phone or digital platforms, could have enhanced participant engagement and motivation. This was particularly relevant during the COVID-19 pandemic, when most individuals were homebound and poorer dietary habits have been reported [36]. The involvement of multiple dietitians in this study was intended to replicate real-world outpatient diabetes practice. However, this approach may have inadvertently undermined the consistency in the interventions. Although feasibility testing was conducted with a group of adults with T1D using MDIs insulin therapy prior to study, greater investment in user involving in the development of the study interventions might have further improved adherence and, potentially, glycemic outcomes.

Another notable limitation is the use of MAGEs as a primary outcome for assessing glycemic variability. When this study was designed in 2017, MAGEs was more commonly used in research settings in Denmark. However, in recent years, international guidelines have recommended time in range as a more reliable and clinically relevant metric for evaluating glycemic control in both research and clinical contexts, especially with the widespread use of CGM and FGM devices in individuals with T1D [25,37,38].

Moreover, this study’s design, which predates substantial advancements in diabetes technology, may not fully represent current clinical practices. At the study’s outset, only 55% of participants were using CGM or FGM devices for diabetes self-management. By August 2024, however, 95% of adults with T1D in our clinic were utilizing glucose sensor technology. Nationally, the adoption rate has also risen, though it remains lower, with approximately 60% of adults with T1D currently using such devices [39]. These technological advancements have markedly altered how diabetes is managed, particularly through the utilization of real-time glucose data for more precise insulin dose adjustments. The widespread use of glucose sensors has independently improved diabetes self-management glycemic outcomes [40]. Consequently, dietary education programs, such as those employed in this study, may need to be adapted to incorporate these modern technological tools and practices.

In addition, the growing integration of advanced diabetes technologies, and artificial intelligence (AI), into the management of T1D presents considerable potential for improving glycemic control through decision support systems that optimize automated insulin therapy [41]. Recent advancements, including automated insulin delivery (AID) systems and more sophisticated insulin pumps, are reshaping how insulin is administered and how dietary education should be approached in clinical settings. However, while AID systems enable individuals with difficulties in precise carbohydrate counting to achieve better glycemic outcomes, higher accuracy in carbohydrate estimation remains crucial for optimal glycemic control [42]. At the same time, AI-driven food recognition systems, though not yet implemented at a national level, are steadily evolving, with the potential to improve carbohydrate estimation accuracy based on meal images [43,44]. Future educational programs should incorporate these technological advancements, utilizing AI and digital health tools to facilitate more personalized and adaptive approaches to insulin therapy. Furthermore, large-scale diabetes data ecosystems, integrating data from multiple interoperable devices (CGM, insulin pumps, smart pens, and activity trackers), are increasingly employing AI techniques to personalize therapy and enhance patient outcomes [45].

While our study primarily focused on traditional carbohydrate counting methods and MDIs therapy—which remain prevalent among the population studied—it is clear that future T1D management will necessitate the integration of traditional methods with cutting-edge technological innovations. Recognizing the critical role of AI and connected devices in insulin therapy represents a pivotal shift in diabetes care which should be reflected in ongoing research and clinical practice.

Currently, the ACC and BCC programs have been merged into a standardized dietary care model offered to adults with T1D undergoing MDIs therapy at our clinic. This includes a 3 h group session dedicated to practical carbohydrate counting education (shortened but similar to the original BCC program), followed by another 3 h session focused on accurate insulin dose adjustment (similar to the original ACC program). These sessions cover the calculation of individualized insulin-to-carbohydrate ratios, insulin sensitivity factors, and the use of various bolus calculators based on personal preferences in conjunction with CGM or FGM devices. A follow-up individualized dietary counseling session (45–60 min), preferably conducted digitally, addresses personal challenges in applying these strategies. The implementation of this combined group-based and individualized dietary education model, as exemplified in our current ACC–BCC program, represents an adaptable and cost-effective approach. Preliminary cost estimates suggest that the time and resources required to prepare and educate individuals with T1D on MDIs therapy amount to an average of 2.5 h of a dietitian’s time per patient, consistent across all three original interventions in the study, as well as the newly integrated ACC–BCC program. The cost estimation indicates that tailoring the education to fit patient preferences and needs can be achieved without increasing overall time and resource demands.

## 5. Conclusions

In conclusion, our study found no significant improvements in HbA1c or MAGEs from group-based interventions with practical, interactive education in the ACC or BCC programs compared with individual dietary counseling in adults with longstanding, moderately uncontrolled T1D on MDIs insulin therapy. This may reflect either the absence of a clinical effect or study limitations like the small sample size and adherence challenges. While the findings remain relevant, particularly for populations still utilizing MDIs therapy, the rapid advancements in technology demand continuous updates to dietary education and insulin dosing strategies. Future research should explore how emerging tools such as AI and automated systems can enhance carbohydrate counting and insulin therapy, ensuring that clinical practices evolve alongside technological innovations in diabetes management, while also recognizing the critical importance of patient engagement and preferences in optimizing outcomes in everyday diabetes care.

## Figures and Tables

**Table 1:** Baseline characteristics.

Characteristics	Overall (*n* = 53 *)	BCC (*n* = 18 *)	ACC (*n* = 18 *)	Standard (*n* = 17 *)
Age (years)	44 (38, 53)	44 (39, 53)	46 (39, 54)	44 (32, 50)
Men, *n* (%)	37 (70)	12 (67)	13 (72)	12 (71)
Caucasian origin, *n* (%)	52 (98)	18 (100)	18 (100)	16 (94)
Diabetes duration (years)	17 (10, 28)	16 (9, 28)	22 (12, 32)	16 (12, 22)
Smoking, *n* (%)				
Current smoker	11 (21)	4 (22)	4 (22)	3 (18)
Previous smoker	18 (34)	4 (22)	9 (50)	5 (30)
Number of smoking years	20 (15, 23)	18 (13, 21)	20 (15, 25)	17 (15, 23)
Education, *n* (%)				
Elementary school	1 (2)	0	0	1 (6)
Upper secondary education	4 (8)	0	1 (6)	3 (18)
Vocational	15 (28)	4 (22)	5 (28)	6 (35)
Short further (<3 y)	4 (8)	2 (11)	2 (11)	0
Medium further (3–4 y)	14 (26)	5 (28)	5 (28)	4 (24)
Long further (>4 y)	15 (28)	7 (39)	5 (28)	3 (18)
Living situation, *n* (%)				
Alone ^1^	17 (32)	6 (33)	6 (33)	5 (30)
With partner ^1^	36 (68)	12 (67)	12 (67)	12 (70)
Glycemic control				
HbA1c, mmol/mol	64 (58, 69)	66 (57, 68)	65 (62, 70)	62 (57, 70)
HbA1c, %	8.0 (7.5, 8.5)	8.2 (7.4, 8.4)	8.1 (7.8, 8.6)	7.8 (7.4, 8.6)
MAGE, mmol/L	6.2 (5.3, 8.1)	5.9 (5.4, 8.2)	6.5 (4.8, 8.0)	6.2 (5.1, 7.8)
Mean p-glucose, mmol/L	9.4 (8.2, 10.3)	9.3 (7.8, 10.4)	9.3 (8.9, 9.9)	9.5 (7.9, 10.3)
CV, %	37.2 (33.8, 43.0)	36.9 (35.3, 41.8)	39.8 (34.8, 42.2)	37.9 (33.7, 44.6)
SD, mmol/L	3.7 (3.1, 4.3	3.4 (2.9, 4.2)	3.6 (3.2, 4.3)	3.7 (3.5, 4.3)
TIR: % time spent 3.9−10.0 mmol/L	56.0 (44.8, 64.7)	56.3 (44.9, 67.3)	54.2 (46.6, 63.0)	56.0 (43.2, 65.2)
TAR: % time spent 10.1−13.9 mmol/L	37.9 (27.1, 49.3)	39.9 (24.2, 47.2)	37.3 (32.4, 50.2)	36.5 (26.2, 49.3)
TBR: % time spent 3.0–3.8 mmol/L	3.4 (0.6, 8.5)	3.8 (0.7, 8.5)	2.1 (0.7, 7.3)	4.8 (0.5, 8.6)
Body weight, kg	81.0 (73.0, 90.6)	83.2 (75.9, 94,3)	78.1 (73.2, 83.0)	79.2 (72.5, 90.6)
BMI, kg/m^2^	26.3 (24.7, 27.8)	27.0 (25.3, 29.2)	25.4 (23.3, 26.9)	26.5 (25.2, 29.3)
Waist/Hip ratio, unitless	0.98 (0.92, 1.03)	0.99 (0.95, 1.04)	0.99 (0.92, 1.03)	0.98 (0.91, 1.02)
Systolic pressure, mmHg	126 (116, 135)	129 (119, 138)	116 (112, 131)	127 (123, 134)
Diastolic pressure, mmHg	76 (71, 84)	78 (74, 84)	72 (68, 79)	78 (73, 88)
LDL cholesterol, mmol/L	2.4 (1.9, 2.9)	2.7 (2.3, 2.9)	2.2 (1.9, 2.6)	2.5 (1.8, 2.9)
Open CGM/FGM users, *n*	29 (55)	9 (50)	12 (67)	8 (47)
Medication				
Basal insulin, units/day	22 (18, 28)	22 (18, 25)	22 (16, 26)	23 (19, 32)
Prandial insulin, units/day	24 (18, 32)	29 (21, 32)	24 (13, 28)	21 (12, 34)
GLP−1Ras, *n* (%)	3 (6)	1 (6)	1 (6)	1 (6)
Antihypertensives, *n* (%)	13 (25)	6 (33)	3 (17)	4 (24)
Lipid-lowering drugs, *n* (%)	17 (32)	5 (28)	8 (44)	4 (24)
CGM/FGM users, *n* (%)	29 (55)	9 (50)	12 (66)	8 (47)

* Dropouts before baseline measurements are not included: BCC (*n* = 2), ACC (*n* = 3), and standard (*n* = 5). ^1^ With or without children living at home. Data are medians (25th and 75th percentiles). Categorical data are summarized by numbers and percentages. Abbreviations: ACC, advanced carbohydrate counting; BCC, basic carbohydrate counting; BMI, body mass index; CGM, continuous glucose monitoring; CV, coefficient of variation; FGM, Flash glucose monitoring; GLP−1RAs, glucagon-like peptide 1 receptor agonists; HbA1c, glycated hemoglobin A1c; LDL, low-density lipoprotein; MAGEs, mean amplitude of glycemic excursions; SD, standard deviation; Standard, standard dietary care; TAR, time above range; TBR, time below range; TIR, time in range.

**Table 2:** Baseline-adjusted estimates for primary and secondary outcomes.

Outcome	Group	Visit	Estimated Mean (95% CI)	Within-Group Changes (95% CI)	Diff. from Control (95% CI)	*p*-Value
HbA1c, mmol/mol	ALL	Baseline	65 (63: 68)			
	Standard	Week 12	63 (60: 67)	−2 (−5: 1)		
		EOT	62 (59: 65)	−3 (−6: −0)		
		Follow-up	61 (57: 66)	−4 (−8: 0)		
	BCC	Week 12	62 (59: 66)	−3 (−6: −0)	−1 (−5: 3)	0.563
		EOT	63 (60: 66)	−2 (−5: 0)	1 (−3: 5)	0.663
		Follow-up	61 (57: 65)	−4 (−8: −0)	−0 (−6: 6)	0.976
	ACC	Week 12	65 (62: 68)	−0 (−3: 2)	2 (−2: 5)	0.415
		EOT	62 (59: 65)	−4 (−6: −1)	−1 (−4: 3)	0.779
		Follow-up	63 (58: 67)	−3 (−7: 1)	1 (−4: 7)	0.653
HbA1c, %	ALL	Baseline	8.1 (7.9: 8.4)			
	Standard	Week 12	7.9 (7.6: 8.3)	−0.2 (−0.5: 0.1)		
		EOT	7.8 (7.6: 8.1)	−0.3 (−0.6: −0.0)		
		Follow-up	7.7 (7.4: 8.2)	−0.4 (−0.7: 0.0)		
	BCC	Week 12	8.2 (7.6: 8.2)	−0.3 (−0.6: −0.0)	−0.1 (−0.5: 0.3)	0.563
		EOT	7.9 (7.6: 8.2)	−0.2 (−0.5: 0.0)	0.1 (−0.3: 0.5)	0.663
		Follow-up	7.7 (7.4: 8.1)	−0.4 (−0.7: −0.0)	−0.0 (−0.6: 0.6)	0.976
	ACC	Week 12	8.1 (7.8: 8.4)	−0.0 (−0.3: 0.2)	0.2 (−0.2: 0.5)	0.415
		EOT	8.2 (7.6: 8.1)	−0.4 (−0.6: −0.1)	−0.1 (−0.4: 0.3)	0.779
		Follow-up	7.9 (7.5: 8.3)	−0.3 (−0.6: 0.1)	0.1 (−0.4: 0.6)	0.653
MAGEs, mmol/L	ALL	Baseline	9.0 (8.4: 9.7)			
	Standard	EOT	8.3 (7.3: 9.4)	−0.7 (−1.8: 0.4)		
	BCC	EOT	8.7 (7.6: 9.8)	−0.3 (−1.5: 0.8)	0.4 (−1.1: 1.9)	0.590
	ACC	EOT	9.0 (7.9: 10.1)	−0.0 (−1.2: 1.1)	0.7 (−0.8: 2.1)	0.360
TIR, %	ALL	Baseline	56.2 (51.6: 60.8)			
	Standard	EOT	54.1 (46.7: 61.6)	−2.1 (−9.3: 5.2)		
	BCC	EOT	55.4 (47.7: 63.0)	−0.8 (−8.4: 6.7)	1.2 (−9.0: 11.4)	0.809
	ACC	EOT	54.8 (47.3: 62.3)	−1.4 (−8.8: 6.0)	0.7 (−9.4: 10.7)	0.893
TBR, % *	ALL	Baseline	3.6 (2.4: 5.3)			
	Standard	EOT	5.4 (2.2: 13.1)	49.5 (−40.9: 278.7)		
	BCC	EOT	2.6 (1.0: 6.7)	−26.7 (−72.3: 93.7)	−51.0 (−86.6: 79.6)	0.271
	ACC	EOT	3.3 (1.4: 7.7)	−8.4 (−62.5: 123.6)	−38.7 (−82.3: 111.9)	0.427
TAR, %	ALL	Baseline	38.4 (33.7: 43.1)			
	Standard	EOT	38.9 (29.9: 47.9)	0.4 (−8.3: 9.1)		
	BCC	EOT	39.2 (29.9: 48.6)	0.8 (−8.2: 9.9)	0.4 (−12.0: 12.8)	0.950
	ACC	EOT	38.1 (29.1: 47.2)	−0.3 (−9.1: 8.6)	−0.7 (−12.9: 11.5)	0.907
Mean PG, mmol/L	ALL	Baseline	9.4 (9.0: 9.9)			
	Standard	EOT	9.3 (8.4: 10.2)	−0.1 (−1.0: 0.7)		
	BCC	EOT	9.4 (8.5: 10.4)	0.0 (−0.9: 0.9)	0.2 (−1.1: 1.4)	0.804
	ACC	EOT	9.3 (8.4: 10.2)	−0.1 (−1.0: 0.8)	0.0 (−1.2: 1.3)	0.939
Basal insulin dose, units/day *	ALL	Baseline	23 (20: 25)			
	Standard	EOT	22 (20: 25)	−2 (−8: 4)		
		Follow-up	22 (20: 26)	−1 (−8: 7)		
	BCC	EOT	23 (20: 26)	−0 (−6: 6)	2 (−6: 11)	0.691
		Follow-up	23 (20: 26)	−0 (−7: 7)	0 (−9: 11)	0.935
	ACC	EOT	23 (20: 26)	0 (−6: 6)	2 (−6: 11)	0.62
		Follow-up	23 (20: 26)	2 (−5: 10)	3 (−7: 14)	0.568
Prandial insulin dose, units/day *	ALL	Baseline	23 (20: 28)			
	Standard	EOT	25 (22: 30)	9 (−6: 26)		
		Follow-up	26 (22: 32)	13 (−5: 35)		
	BCC	EOT	26 (22: 31)	12 (−4: 29)	2 (−15: 24)	0.805
		Follow-up	27 (23: 33)	17 (−1: 38)	3 (−18: 30)	0.770
	ACC	EOT	27 (23: 31)	14 (−1: 32)	5 (−13: 26)	0.614
		Follow-up	30 (25: 36)	27 (7: 51)	12 (−11: 41)	0.317
TTD insulin, units/day *	ALL	Baseline	47 (42: 54)			
	Standard	EOT	49 (43: 56)	3 (−4: 12)		
		Follow-up	51 (44: 58)	7 (−4: 18)		
	BCC	EOT	49 (43: 56)	4 (−4: 12)	0 (−10: 12)	0.954
		Follow-up	51 (44: 58)	6 (−3: 17)	−0 (−13: 14)	0.971
	ACC	EOT	50 (44: 57)	5 (−3: 14)	2 (−8: 14)	0.710
		Follow-up	54 (47: 61)	13 (2: 25)	6 (−8: 22)	0.404
Median carb estimation errors, g *	ALL	Baseline	33.3 (17.9: 62.0)			
	Standard	EOT	24.9 (12.8: 48.7)	−25.0 (−63.6: 54.4)		
		Follow-up	13.1 (6.1: 28.1)	−60.7 (−83.7: −5.1)		
	BCC	EOT	8.7 (4.4: 17.3)	−73.8 (−88.0: −42.8)	−65.0 (−86.1: −12.0)	0.028
		Follow-up	13.1 (5.5: 31.5)	−60.5 (−85.5: 7.2)	0.5 (−68.7: 222.7)	0.993
	ACC	EOT	8.7 (4.0: 19.1)	−73.8 (−88.8: −38.8)	−65.0 (−87.1: −5.4)	0.040
		Follow-up	10.4 (4.3: 25.1)	−68.6 (−88.2: −16.3)	−20.1 (−75.1: 156.5)	0.692
Median carb estimation errors, % *	ALL	Baseline	157 (120: 204)			
	Standard	EOT	136 (108: 170)	−13 (−35: 16)		
		Follow-up	126 (100: 158)	−20 (−42: 11)		
	BCC	EOT	119 (95: 149)	−24 (−43: 1)	−12 (−35: 19)	0.400
		Follow-up	117 (94: 145)	−26 (−46: 2)	−7 (−32: 27)	0.636
	ACC	EOT	105 (84: 131)	−33 (−49: −11)	−23 (−43: 5)	0.096
		Follow-up	116 (93: 145)	−26 (−46: 1)	−8 (−33: 27)	0.619

Estimated means (CI 95%) (left) and baseline corrected difference between groups (CI 95%). *p*-values are between group differences. * Variable has been log-transformed for analysis and back−transformed for presentation. Abbreviations: ACC, advanced carbohydrate counting; BCC, basic carbohydrate counting; EOT, end-of-treatment at six months; HbA1c, hemoglobin A1c; MAGEs, mean amplitude of glycemic excursions; PG, plasma glucose; Standard, standard dietary treatment; TAR time above range (>10.0 mmol/L); TBR, time below range (<3.9 mmol/L); TIR, time in range (3.9−10.0 mmol/L), TTD, total daily dose.

## Data Availability

The data presented in this study are available on request from the corresponding author due to confidentiality concerns.

## Supplementary Materials

The following supporting information can be downloaded at: https://www.mdpi.com/article/10.3390/nu16213745/s1, File S1: Statistical Analyses Plan (SAP), Figure S1: Flow diagram; Table S1: Selected variables for dropouts before baseline measurements vs. overall sample; Table S2: Baseline characteristics supplementary; Table S3: Participants prescribed GLP−1Ras, antihypertensive, and lipid-lowering medication at baseline, end-of-treatment, and follow-up; Table S4: Mealtime insulin adjustments at baseline, end-of-treatment, and follow-up; Table S5: Baseline-adjusted estimates for primary and secondary/exploratory outcomes—supplementary; Table S6: Delta values for diabetes diet-related quality of life, perceived autonomy support, and competencies in diet and diabetes.
