# Supplementary material for: Comparing the Effectiveness of Different Dietary Educational Approaches for Carbohydrate Counting on Glycemic Control in Adults with Type 1 Diabetes: Findings from the DIET-CARB Study, a Randomized Controlled Trial
Source: Nutrients. 2024 Oct 31;16(21):3745. doi: 10.3390/nu16213745 (PMC11547945; doi:10.3390/nu16213745)
Supplement: Supplementary file 1 [file nutrients-16-03745-s001.zip › File S1. SAP.pdf]

Statistical Analysis Plan for the manuscript reporting primary and selected secondary outcomes from the DIET-CARB study

Official title of the study:

The Dietary Education Trial in Carbohydrate Counting (DIET-CARB Study): A randomized, parallel, open-label, intervention study comparing different approaches to dietary self-management in patients with type 1 diabetes

ClinicalTrials.gov ID: NCT03623113

Date of document: 2 January 2024

Contents

Section 1: Administrative information ... 3
Signature page ... 4
Section 2: Introduction ... 5
Background and Rationale (adapted from study protocol) ... 5
Objectives ... 6
Section 3: Study Methods ... 7
Trial design ... 7
Randomisation ... 7
Sample size ... 7
Framework ... 7
Statistical interim analyses and stopping guidance ... 7
Timing of final analyses ... 7
Timing of outcome assessments ... 7
Section 4: Statistical Principles ... 8
Confidence intervals and P values ... 8
Completers and protocol deviations ... 8
Analysis populations ... 8
Section 5: Trial Population ... 9
Screening data ... 9
Eligibility ... 9
Recruitment ... 9
Withdrawal/follow-up ... 9
Baseline participant characteristics ... 9
Section 6: Analysis ... 11
Outcome definitions ... 11
Analysis methods ... 14
Missing data ... 14
Additional analyses ... 14
Harms ... 14
Statistical software ... 14
References ... 15

Section 1: Administrative information

Title: *The Dietary Education Trial in Carbohydrate Counting (DIET-CARB Study): A randomized, parallel, open-label, intervention study comparing different approaches to dietary self-management in patients with type 1 diabetes*

Publication date: 2 January 2024
SAP version: 1
Protocol version: 8
ClinicalTrials.gov ID: NCT03623113
Journal no.: H-18014897 (The study has been approved by the Danish ethics committee in Copenhagen).

This document is a supplement to the DIET-CARB study protocol (1). The document contains the statistical analysis plan (SAP) for the main paper of the trial in which the primary outcomes and selected secondary outcomes will be reported. This document complies with the guidelines for content of statistical analysis plans in clinical trials (2).

Principal investigator Bettina Ewers, MSc PhD
Steno Diabetes Center Copenhagen
Borgmester Ib Juuls Vej 83
DK-2730 Herlev
Denmark
E-mail: bettina.ewers@regionh.dk
Phone: +45 3091 2997

Signature page

To be signed by individual writing the Statistical Analysis Plan (SAP), the statistical advisor, contributors to the SAP, principal investigator, and co-investigators.

Title: *The Dietary Education Trial in Carbohydrate Counting (DIET-CARB Study): A randomized, parallel, open-label, intervention study comparing different approaches to dietary self-management in patients with type 1 diabetes*

ClinicalTrials.gov ID: NCT03623113

Name	Title	Role	Signature	Date
Bettina Ewers[1]	MSc, PhD	Principal investigator, data analyst, SAP writer	Bettina Ewers	22.12.23
Martin Bæk Blond[1]	MSc, PhD	Statistical advisor, contributor to SAP	[illegible]	22-12-23
Jens Meldgaard Bruun[2,3]	MD, PhD	Co-investigator	[illegible]	19/12-23
Tina Vilsbøll[1,4]	MD DMSc	Co-investigator	Tina Vilsbøll	19/12-23

Affiliations

1. Steno Diabetes Center Copenhagen, Herlev, Denmark
2. Steno Diabetes Center Aarhus, Aarhus, Denmark
3. Department of Clinical Medicine, University of Aarhus, Denmark
4. Faculty of Health and Medical Sciences, University of Copenhagen, Denmark

Section 2: Introduction

Background and Rationale (adapted from study protocol)

The total amount of carbohydrates consumed in a meal is a major predictor of the postprandial glucose response (3). Thus, monitoring dietary intake of carbohydrates is important to control postprandial glucose fluctuations in type 1 diabetes (T1D). A reduction in postprandial glucose excursions may in turn lead to clinical benefits such as attenuated glucose variability and improvements of glycated haemoglobin A1c (HbA1c), and potentially to a reduction in diabetes-related complications. Clinical guidelines in medical nutrition therapy recommend that people with T1D learn carbohydrate counting or similar experience-based methods to improve glycaemic control (3-6).

Two levels of carbohydrate counting have been defined with increasing complexity: A basic and an advanced level (7-8). In brief, basic carbohydrate counting (BCC) includes education in estimating total carbohydrate amounts, and improving consistency in the timing, type, amount, and distribution of carbohydrate-containing foods at meals. Advanced carbohydrate counting (ACC) includes education in how to adjust mealtime insulin dose. Ideally, people with T1D treated on multiple daily injections (MDI) therapy need to be able to manage both levels. This includes managing two calculation steps several times daily when using carbohydrate counting: (1) Correct estimation of the total carbohydrate content at each meal (equal to BCC), and (2) Correct estimation of mealtime insulin dose according to the carbohydrate amount consumed at each meal using a carbohydrate-to-insulin ratio, an insulin sensitivity factor, and the current and target plasma glucose (equal to ACC). Thus, good mathematical skills are highly essential. However, studies have found that people with T1D frequently assess their intake of carbohydrates inaccurately and this has been associated with a poorer HbA1c, and higher daily plasma glucose variability (9-12). In clinical guidelines and trials, the term 'carbohydrate counting' is often used synonymously with ACC, while the sole effect of BCC on glycaemic control is largely unknown. Systematic reviews and meta-analyses of randomised controlled trials (RCTs) have shown that ACC can reduce HbA1c by up to 7 mmol/mol in adults with poorly controlled T1D (13-15). Despite this, systematic educating and training in BCC and ACC is still not offered routinely for people with T1D on multiple daily injections (MDI) therapy in outpatient clinics in Denmark. This may be due to the lack of evidence as to which educational methods are the most effective for training people, and the lack of appropriate learning environments to promote behavioural change and availability of trained dietitians to facilitate the learning process. Additionally, the concept of ACC may not apply to all people with T1D on MDI therapy e.g., due to lack of motivation, low literacy, or numeracy skills.

Objectives

The primary objective of the DIET-CARB study is to investigate the effectiveness of two different educational approaches for carbohydrate counting (BCC and ACC) and usual dietary care on change in HbA1c and glycaemic variability (assessed by mean amplitude of glycaemic excursions (MAGE)) in people with T1D.

Secondary objectives were to describe changes associated with the intervention on other clinically relevant metabolic changes (body weight, body composition, blood pressure and lipid profile), changes in skills related to numeracy and carbohydrate estimation accuracy as well as on psychosocial and behavioural outcomes at the end of the intervention period and after 6 months of follow-up to assess long-term maintenance.

Hypotheses for the primary outcomes:

1. We hypothesize that ACC is superior compared with usual dietary care (the control group) in reducing HbA1c or MAGE from baseline to end-of-treatment at week 24 (from V1 to V2).

Superiority is claimed if:

a. the 95% confidence interval for the estimated difference in change between the groups for HbA1c or MAGE, estimated using a baseline corrected linear mixed model, excludes 0 and the P-value is <0.05: and,
b. the estimated difference in HbA1c or MAGE between the two groups is equal to/surpass the minimal important difference in favour of the ACC group.

2. We hypothesize that BCC is superior compared with usual dietary care (control group) in reducing HbA1c or MAGE from baseline to end-of-treatment at week 24 (from V1 to V2).

Superiority is claimed if:

c. the 95% confidence interval for the estimated difference in change between the groups for HbA1c or MAGE, estimated using a baseline corrected linear mixed model, excludes 0 and the P-value is <0.05: and,
d. the estimated difference in HbA1c or MAGE between the two groups is equal to/surpass the minimal important difference in favour of the BCC group.

3. We hypothesize that BCC is equivalent to ACC in reducing HbA1c or MAGE from baseline to end-of-treatment at week 24 (from V1 to V2).

Equivalence is claimed if:

e. The 90% confidence interval for the estimated difference in change between the groups for HbA1c or MAGE does not surpass minimal important difference in a negative or positive direction (16).

Hypotheses for the secondary outcomes can be found in the study protocol and clinicaltrials.gov.

Section 3: Study Methods

Trial design

Single-centre parallel-group, randomised controlled, open-label, superiority trial. Allocation ratio 1:1:1 to either ACC, BCC, or control (usual dietary management).

Randomisation

Participants were randomised using block randomisation by stratifying participants based on sex and HbA1c at baseline. The randomization list was generated by an external statistician and uploaded to the electronic data management system REDCap (8.10.18, Vanderbilt University, TN, USA). Participants eligible for inclusion in the study according to the screening were randomised at the end of the screening visit (V0) by the study investigator/study personnel using the randomization module in REDCap. Baseline measurements were collected at the following baseline visit (V1) for alle participants.

Sample size

See study protocol.

Framework

Superiority trial. See Objectives section.

Statistical interim analyses and stopping guidance

No interim analyses were planned and no guidelines for terminating the trial early was made.

Timing of final analyses

The results will be analysed when this statistical analysis plan has been uploaded at clinicaltrials.gov

Timing of outcome assessments

HbA1c was measured at the screening visit (V0), at the baseline visit (V1), after 12 weeks of intervention, after 24 weeks (V2) of intervention, and after six months follow-up (V3). MAGE was measured after the baseline visit (V1), and after 24 weeks of intervention (after V2).

See section 6: Analyses for timing of secondary/descriptive/explorative outcome assessments.

Section 4: Statistical Principles

Confidence intervals and P values

Two-sided P-values and 95% confidence intervals will be presented for comparisons between groups. Two-sided 95% confidence intervals will be presented for within group comparisons and estimated levels.

Primary outcome: The direction and size of the estimated mean effect for HbA1c and MAGE (primary outcomes), in addition to the 95% confidence intervals, will be required to support the tested hypotheses for the results to be declared in accordance with the hypotheses (see section 2 under "Objectives").

Secondary outcomes: False detection rate (FDR) correction ad modem Benjamini and Hochberg (17) will be used to control for multiplicity; < 5% will be used as the threshold for FDR.

Completers and protocol deviations

Completers: Participants who participated in an assessment of at least one of the primary outcomes at V2 and/or V3.

Lost to follow-up: Participants who did not participate in assessment of primary outcomes at V2.

Excluded for statistical data analyses: Participants in the three study groups dropping out or lost to follow-up before baseline measurements have been collected.

Protocol deviators: None of the participants in the two study groups are considered protocol deviators.

Analysis populations

<u>Efficacy estimates based on intention-to-treat (ITT) analysis set</u>:
All participants will be analysed as randomised.

Section 5: Trial Population

Screening data

The following data obtained at the screening visit will be included for those that entered the trial:

- Age
- Self-reported sex
- Self-reported ethnicity
- Self-reported educational level
- Self-reported smoking status and number of years of smoking
- Diabetes duration

Eligibility

- Age: ≥18 to ≤75 years
- Diabetes duration: >12 months
- HbA1c: 53–97 mmol/mol
- Multiple daily insulin injection therapy
- Diagnosed with T1D and treated in a hospital in the Capital Region of Denmark

Recruitment

The flow diagram of the trial will comply with the most recent CONSORT guidelines for Reporting Outcomes in Trial Reports and includes:

1. Total number assessed for eligibility
2. Total number excluded (numbers not meeting criteria or declined to participate)
3. Total numbers randomised
4. Total number of participants who were randomly allocated to each group
5. The numbers of participants who received and did not receive the allocated treatment in each group
6. The numbers who were analysed for the primary outcomes in each group
8. For each group, losses, and exclusions after randomisation, together with reasons are reported

Withdrawal/follow-up

The level of consent and consent withdrawal will be tabulated. Participants without HbA1c and MAGE measurements at V2 will be regarded as lost-to-follow-up in relation to the primary outcomes during the intervention period and those with a missing HbA1c measurement at V3 will be regarded as lost-to-follow-up in relation to the primary outcome during the follow-up period. The number of participants lost-to-follow-up for each group during each phase of the trial will be reported in the CONSORT diagram. Summary of baseline levels for variables reported in the baseline table will be provided for completers and for those lost to follow-up/discontinuation of intervention after the baseline visit.

Baseline participant characteristics

The distribution of all continuous outcomes included in baseline characteristics will be visually inspected using QQ-plots and histograms; those with a Gaussian distribution will be presented as means and standard deviations and those with a non-Gaussian distribution will be presented as medians plus 25th and 75th percentiles, number of observations will be presented for each outcome presented. Categorical data will be summarised by numbers and percentages. Tests of statistical significance will not be undertaken for baseline

characteristics according to the CONSORT Statement for reporting clinical trials; rather the clinical importance of any imbalance will be noted (18).

The following outcomes will be included in the baseline participant characteristics table for all participants combined and stratified by randomisation group:

- Number of participants, n
- Age (years)
- Gender, male, n and %
- Ethnicity, white, n and %
- Smoking status
    - Current smoker, n and %
    - Number of smoking years
- Education
    - Elementary school, n and %
    - Upper secondary education, n and %
    - Vocational, n and %
    - Short further (< 3 y), n and %
    - Medium further (3-4 y), n and %
    - Long further (> 4 y), n and %
- Family status
    - Living alone with or without children at home, n and %
    - Living with a partner with or without children at home, n and %
- Diabetes duration (years)
- HbA1c (mmol/mol)
- Blinded sensor data
    - MAGE (mmol/l)
    - Mean plasma glucose, mmol/l
    - Time in range (TIR): % of readings and time 3.9-10.0 mmol/l
    - Time above range (TAR): % and readings and time 10.1-13.9 mmol/l
    - Time below range (TBR): % readings and time 3.0-3.8 mmol/l
    - Glycaemic variability, coefficient of variation (CV), %
    - Glycaemic variability, standard deviation (SD), mmol/l
- Body weight (kg)
- BMI, kg/m^2
- Waist/Hip ratio, unitless (women)
- Waist/Hip ratio, unitless (men)
- Systolic blood pressure (mmHg)
- Diastolic blood pressure (mmHg)
- LDL cholesterol (mmol/l)
- Glucose-lowering medication
    - Basal insulin dose, units/day
    - Prandial insulin dose, units/day
    - GLP-1, n and %
- Antihypertensives, n and %
- Lipid-lowering medication, n and %
- Use of flash or continuous glucose monitors, n and %

Section 6: Analysis

Outcome definitions

Primary outcomes

HbA1c and MAGE. The treatment effect will be given as the baseline corrected difference in mmol/mol for HbA1c and mmol/l for MAGE between the ACC versus the control group, and the BCC versus the control group at V2.

Estimation of minimal important difference for primary outcome: Reductions in HbA1c of 3.5 mmol/mol and 0.35 mmol/l of MAGE were defined as minimal important differences in this trial. This was based on findings from experimental studies examining the HbA1c effect of BCC (19-21), and meta-analyses of RCTs examining the HbA1c effect of ACC (13-14) vs. control or usual dietary treatment. In these studies, HbA1c reductions between 3 to 7 mmol/mol had been found in adults with T1D. All participants in the studies had a more poorly controlled diabetes (60-108 mmol/mol) than participants eligible for inclusion in our study (53-97 mmol/mol). Consequently, we expected to find smaller HbA1c reductions in our study population. This was considered clinically relevant as part of a multidisciplinary approach for improving glycaemic control in T1D.

Secondary/descriptive/exploratory outcomes

The treatment effect will be given as the baseline corrected difference between the groups at V2 and V3. No minimal clinically relevant differences were defined for these outcomes.

- HbA1c, mmol/mol (only for V3)
- Blinded CGM data (not measured at V3)
  - Mean amplitude of glycaemic excursions (MAGE), mmol/l
  - Mean plasma glucose, mmol/l
  - Time in range (TIR): % of readings and time 3.9-10.0 mmol/l
  - Time above range (TAR): % of readings and time 10.1-13.9 mmol/l (level 1 – high)
  - Time above range (TAR): % of reading and time >13.9 mmol/l (level 2 – very high)
  - Time above range: % of readings and time >13.9 mmol/l
  - Time below range (TBR): % readings and time 3.0-3.8 mmol/l (level 1 – low)
  - Time below range (TBR): % readings and time <3.0 mmol/l (level 2 – very low)
  - Glycaemic variability, coefficient of variation (CV), %
- BMI (kg/m2)
- Body weight (kg)
- Waist/Hip ratio, unitless
- Blood pressure
  - Systolic blood pressure (mmHg)
  - Diastolic blood pressure (mmHg)
- LDL cholesterol (mmol/l)
- Median carbohydrate estimation errors, g
- Median carbohydrate estimation errors, %
- Self-reported diabetes diet-related quality of life (DDRQOL), total score within each subscale
  - General perception of diet

  - Satisfaction with diet
  - Restriction of social functions
  - Burden of dietary therapy
  - Perceived benefits of dietary therapy
  - Mental health
  - Vitality
- Self-reported Perceived Health Competence in Diabetes Scale (PCDS), total score
- Self-reported Healthcare Climate Questionnaire (HCCQ) (not measured at V3), total score

Other outcomes

- Number of visits with dietitians, endocrinologists, and diabetes nurses at V2 and V3 in each group.
- Use of bolus calculator to assist insulin dose decision-making in the ACC arm collected after V2.
- Self-reported use of tools to estimate carbohydrate amounts in meals for each group at V1, V2, V3.
- Self-reported frequency and method for estimating mealtime insulin dose for each group at V1, V2, V3.
- Supplementary table with baseline characteristics for completers vs. non-completers with dropout before V2
  - Number of participants, n
  - Age (years)
  - Gender, male, n and %
  - Current smokers, n and %
  - Self-reported educational level
    - Elementary school, n and %
    - Upper secondary education, n and %
    - Vocational, n and %
    - Short further (< 3 y), n and %
    - Medium further (3-4 y), n and %
    - Long further (> 4 y), n and %
    - Other education, n and %
  - Living alone, n and %
  - Body weight (kg)
  - BMI (kg/m2)
  - Diabetes duration (years)
  - HbA1c (mmol/l)
- Supplementary table with extra baseline characteristics
  - Occupation
    - Employed/self-employed, n and %
    - Unemployed / job seeking, n and %
    - Retired, n and %
    - Other (on leave / studying), n and %
  - Annual household income (before tax)
    - < 100,000 DKK (<13,500 EUR)
    - 100,000 – 200,000 DKK (13,500 – 27,000 EUR)
    - 200,000 – 400,000 DKK (27,000 – 54,000 EUR)
    - 400,000 – 600,000 DKK (54,000 – 81,000 EUR)
    - 600,000 – 800,000 DKK (81,000 – 108,000 EUR)
    - 800,000 DKK (>108,000 EUR)
    - Unspecified
  - Waist circumference, cm (women)
  - Waist circumference, cm (men)

- Fasting concentration of plasma lipids
    - Total cholesterol (mmol/l)
    - HDL cholesterol (mmol/l)
    - Triglycerides (mmol/l)
- Dietary intake
    - Total energy, kJ/day (women)
    - Total energy, kJ/day (men)
    - Carbohydrates, g/day (men)
    - Carbohydrates, g/day (women)
    - Carbohydrates, E%
- Physical activity level
    - Low, n and %
    - Moderate, n and %
    - High, n and %

• Supplementary table with baseline-adjusted estimates for other secondary/exploratory outcomes
- Glycaemic variability, standard deviation (SD), mmol/l
- Average waist, cm
- Fasting concentration of plasma lipids
    - Total cholesterol (mmol/l)
    - HDL cholesterol (mmol/l)
    - Triglycerides (mmol/l)
- Numeracy skills test, correct answers, %
- Dietary intake (not measured at V3)
    - Total energy intake (kJ/day)
    - Carbohydrate intake (E%)
    - Carbohydrate intake (g/day)
    - Total fat (E%)
    - Protein (E%)
    - Saturated fat (g/day)
    - Monounsaturated fat (g/day)
    - Polyunsaturated fat (g/day)
    - Dietary fibre (g/day)
    - Dietary fibre (g/10 MJ)
    - Added sugar (g/day)
    - Added sugar (g/10 MJ)
- Self-reported physical activity (International Physical Activity Questionnaire – Short Form (IPAQ SF))
    - MET-minutes/week

• Supplementary table with delta values for diabetes diet-related quality of life (DDQOL), perceived autonomy support (HCCQ) and competencies in diet and diabetes scale (PCDS)
- DDQOL subscales (score)
    - Dietary satisfaction
    - Dietary benefits
    - Dietary burden
    - Social restrictions
    - General perception of diet
    - Mental health
    - Vitality
- PCDS (total score)
- HCCQ (total score)

Analysis methods

Analyses of the primary outcomes will be performed based on efficacy estimates.

Before further analysis and before unblinding, all variables will be inspected to detect outliers to uncover potential errors, such as registration errors.

All continuous outcomes covered by this SAP will as a rule be modelled using baseline corrected repeated measures regression (22) with the following fixed effects and interactions between fixed effects: Visit, Visit (factorial)*Treatment. Data from V1, V2, and V3 will be included in the analysis. The models will be specified with a restricted maximum likelihood estimation method and a repeat on participant level (unstructured covariance structure). Model fit will be evaluated using graphical methods before estimating the treatment effects and if necessary, outcomes will be log-transformed. Estimated mean differences (CI95%) between groups (CI95%), conditional means (CI95%), and within group changes (CI95%) will be extracted from the model. For log-transformed outcomes the results will be back-transformed and be presented as the ratio between estimated mean differences (CI95%), estimated conditional geometric means (CI95%) and relative changes within groups (CI95%), respectively. If log-transformation cannot meet distribution assumptions, a generalized mixed linear model with an appropriate distribution will be applied instead of the repeated measures regression model. In case the distribution does not comply with the distributions available in the generalized mixed linear model a non-parametric test will be used to compare the change scores for the given outcome.

Missing data

The number/frequency of missing values for the primary outcomes in each group at each time point will be provided. In the primary analysis, missing data are handled implicitly by maximum likelihood estimation in the linear mixed model and missing data will be assumed to be missing at random. This is equivalent to making multiple imputations for each treatment group separately and estimating the treatment effect that would have been found had all subject completed their assigned treatment (efficacy estimate) under the missing at random assumption.

Additional analyses

Not relevant.

Harms

Data on harms are not systematically collected and will not be reported.

Statistical software

SAS Enterprise Guide software version 8.3 or newer (SAS Institute Inc., Cary, NC, USA) and R software version 4.0.2 or newer (R Core Team, R Foundation for Statistical Computing, Vienna, Austria.

References

1. Ewers B, Vilsbøll T, Andersen HU, Bruun JM. The dietary education trial in carbohydrate counting (DIET-CARB Study): study protocol for a randomised, parallel, open-label, intervention study comparing different approaches to dietary self-management in patients with type 1 diabetes. BMJ Open 2019;9:e029859. doi:10.1136/bmjopen-2019-029859
2. Gamble C, Krishan A, Stocken D, Lewis S, Juszczak E, Doré C et al. Guidelines for the content of statistical analysis plans in clinical trials. JAMA 2017; 318: 2337–2343
3. Sheard NF, Clark NG, Brand-Miller JC, Franz MJ, Pi-Sunyer FX, Mayer-Davis E, et al. Dietary carbohydrate (amount and type) in the prevention and management of diabetes: a statement by the American Diabetes Association. Diabetes Care. 2004; 27:9:2266-71.
4. Evert AB, Boucher JL, Cypress M, Dunbar SA, Franz MJ, Mayer-Davis EJ, et al. Nutrition therapy recommendations for the management of adults with diabetes. Diabetes Care. 2013;36(11):3821-42.
5. Kliniske retningslinjer for behandling af voksne med Type 1 diabetes. Sundhedsstyrelsen (Danish Health & Medicines Authority), Copenhagen; 2010.
6. Rammeplan for diætbehandling af type 1 diabetes Foreningen af kliniske diætister (The association of clinical dietitians); 2011.
7. Gillespie SJ, Kulkarni KD, Daly AE. Using carbohydrate counting in diabetes clinical practice. J Am Diet Assoc 1998; 98:897–905.
8. Kulkarni KD. Carbohydrate counting: a practical Meal-Planning option for people with diabetes. Clinical Diabetes 2005; 23:120–2.
9. Bishop FK, Maahs DM, Spiegel G, et al. The carbohydrate counting in adolescents with type 1 diabetes (CCAT) study. Diabetes Spectrum 2009; 22:56–62.
10. Mehta SN, Quinn N, Volkening LK, et al. Impact of carbohydrate counting on glycemic control in children with type 1 diabetes. Diabetes Care 2009; 32:1014–6.
11. Smart CE, Ross K, Edge JA, et al. Can children with type 1 diabetes and their caregivers estimate the carbohydrate content of meals and snacks? Diabet Med 2010; 27:348–53.
12. Brazeau AS, Mircescu H, Desjardins K, et al. Carbohydrate counting accuracy and blood glucose variability in adults with type 1 diabetes. Diabetes Res Clin Pract 2013; 99:19–23
13. Bell KJ, Barclay AW, Petocz P, et al. Efficacy of carbohydrate counting in type 1 diabetes: a systematic review and meta-analysis. The Lancet Diabetes & Endocrinology 2014; 2:133–40.
14. Fu S, Li L, Deng S, et al. Effectiveness of advanced carbohydrate counting in type 1 diabetes mellitus: a systematic review and metaanalysis. Sci Rep 2016; 6:37067.
15. Schmidt S, Schelde B, Nørgaard K. Effects of advanced carbohydrate counting in patients with Type 1 diabetes: a systematic review. Diabet. Med. 2014; 31:886–96.
16. Rogers JL, Howard KI, Vessey JT. Using significance tests to evaluate equivalence between two experimental groups. Psychological Bulletin 1993; 113: 3: 553–565. https://doi.org/10.1037/0033-2909.113.3.553
17. Benjamini Y, Hochberg Y. Benjamini Y, Hochberg Y. Controlling the false discovery rate: a practical and powerful approach to multiple testing. J R Stat Soc B 1995; 57: 289–300.
18. Butcher NJ, Monsour A, Mew EJ. Guidelines for Reporting Outcomes in Trial Reports The CONSORT-Outcomes 2022 Extension. Clinical Review and Education. JAMA Special Communication. JAMA 2022: 328:22:2252-2264
19. Scavone G, Manto A, Pitocco D, Gagliardi L, Capoto S, Mancini L, Zaccardi F, Ghirlanda G. Effect of carbohydrate counting and medical nutritional therapy on glycaemic control in Type 1 diabetes subjects: a pilot study. Diabetic medicine 2010; 477-479.
20. Souto DL, Zajdenverg L, Rodacki M, Rosado EL. Impact of advanced and basic carbohydrate counting methods on metabolic control in patients with type 1 diabetes. Nutrition 2014; 286-290.

21. Dias VM, Pandini JA, Nunes RR, Sperandei SLM, Portella ES, Cobas RA, Gomes MdeB. Effect of the carbohydrate counting method on glycemic control in patients with type 1 diabetes. Diabetology & metabolic syndrome 2010; 2:54
22. Liu GF, Lu K, Mogg R, Mallick M, Mehrotra D V. Should baseline be a covariate or dependent variable in analyses of change from baseline in clinical trials? Stat Med 2009; 28: 2509-2530.
