# Supplementary material for: Comparing the Effectiveness of Different Dietary Educational Approaches for Carbohydrate Counting on Glycemic Control in Adults with Type 1 Diabetes: Findings from the DIET-CARB Study, a Randomized Controlled Trial
Source: Nutrients. 2024 Oct 31;16(21):3745. doi: 10.3390/nu16213745 (PMC11547945; doi:10.3390/nu16213745)
Supplement: Supplementary file 1 [file nutrients-16-03745-s001.zip › Table S1. Characteristics of dropouts before baseline.pdf]

Table S1 Selected variables for dropouts before baseline measurements vs. overall sample

	**Dropouts before baseline visit** ***(n = 10)***	**Participants included for analyses** ***(n = 53)***
Age (years)	46 (35, 54)	44 (38, 53)
Women, n (%)	6 (60)	16 (30)
Diabetes duration (years)	15 (8, 21)	17 (10, 28)
Body weight (kg)	70.6 (66.6, 79.6)	81.0 (73.0, 90.6)
BMI (kg/m^2)	26.1 (23.3, 29.3)	26.3 (24.7, 27.8)
HbA1c (mmol/mol)	67 (59, 78)	64 (58, 69)
Education, n (%)		
Elementary school	2 (20)	1 (2)
Upper secondary education	2 (20)	4 (8)
Vocational	1 (10)	15 (28)
Short further (<3 y)	2 (20)	4 (8)
Medium further (3-4 y)	-	14 (26)
Long further (>4 y)	3 (30)	15 (28)
Occupation, n (%)		
Employed/self-employed	7 (70)	39 (74)
Unemployed/job seeking	-	6 (11)
Retired and other*	3 (30)	7 (13)
Unspecified	-	1 (2)
Smoking, n (%)		
Current smoker	2 (20)	11 (21)
Previous smoker	5 (50)	18 (34)

*Other defined as on leave/studying. Data are medians (25th and 75th percentiles). Categorical data are summarized by numbers and percentages. Abbreviations: BMI, body mass index; HbA1c, glycated hemoglobin A1c.
