# Supplementary material for: Comparing the Effectiveness of Different Dietary Educational Approaches for Carbohydrate Counting on Glycemic Control in Adults with Type 1 Diabetes: Findings from the DIET-CARB Study, a Randomized Controlled Trial
Source: Nutrients. 2024 Oct 31;16(21):3745. doi: 10.3390/nu16213745 (PMC11547945; doi:10.3390/nu16213745)
Supplement: Supplementary file 1 [file nutrients-16-03745-s001.zip › Table S2. Baseline supplementary data.pdf]

Table S2 Baseline characteristics – supplementary

**Characteristics**	**Overall** *(n = 53)*	**BCC** *(n = 18)*	**ACC** *(n = 18)*	**STANDARD** *(n = 17)*
Occupation, n (%):				
Employed/self-employed	39 (74)	13 (72)	12 (67)	14 (82)
Unemployed/job seeking	6 (11)	3 (17)	3 (17)	-
Retired and other*	7 (13)	1 (6)	3 (17)	3 (18)
Unspecified	1 (2)	1 (6)	-	-
Annual household income (before tax)				
< 100,000 DKK (<13,500 EUR)	-	-	-	-
100,000 – 200,000 DKK (13,500 – 27,000 EUR)	4 (8)	-	2 (11)	2 (12)
200,000 – 400,000 DKK (27,000 – 54,000 EUR)	8 (15)	2 (11)	3 (17)	3 (18)
400,000 – 600,000 DKK (54,000 – 81,000 EUR)	10 (19)	4 (22)	1 (6)	5 (29)
600,000 – 800,000 DKK (81,000 – 108,000 EUR)	7 (13)	4 (22)	-	3 (18)
800,000 DKK (>108,000 EUR)	17 (32)	6 (33)	8 (44)	3 (18)
Unspecified	7 (13)	2 (11)	4 (22)	1 (6)
Waist circumference, cm (men)	100.0 (88.3, 103.5)	100.0 (90.8, 103.3)	89.8 (88.5, 97.5)	96.8 (86.8, 104.0)
Waist circumference, cm (women)	83.5 (80.3, 88.6)	85.4 (82.0, 89.5)	83.5 (79.0, 92.5)	79.5 (77.8, 86.5)
Fasting plasma lipids				
Total cholesterol, mmol/l	4.4 (4.0, 4.9)	4.6 (4.3, 4.9)	4.1 (4.0, 4.6)	4.5 (3.8, 4.8)
HDL cholesterol, mmol/l	1.5 (1.2, 1.8)	1.6 (1.2, 1.9)	1.5 (1.4, 1.8)	1.4 (1.2, 1.6)
Triglycerides, mmol/l	0.9 (0.6, 1.0)	0.9 (0.7, 1.3)	0.9 (0.7, 1.0)	0.8 (0.6, 1.2)
Dietary intake				
Total energy, kJ/day	8374 (6946, 9645)	7485 (7157, 9077)	8437 (6443, 9576)	8396 (7586, 10218)
Carbohydrates, g/day	187 (162, 225)	191 (167, 208)	187 (157, 204)	184 (172, 258)
Carbohydrates, E%	42 (39, 47)	43 (41, 47)	41 (38, 46)	42 (41, 50)
Median carbohydrate estimation errors, %	114 (100, 240)	127 (100, 237)	112 (96, 286)	129 (100, 193)
Physical activity level, n (%)				
Low	14 (26)	5 (28)	4 (22)	5 (29)
Moderate	17 (32)	6 (33)	4 (22)	7 (41)
High	22 (42)	7 (39)	10 (56)	5 (29)

*Other defined as on leave/studying. Data are medians (25th and 75th percentiles). Categorical data are summarized by numbers and percentages. Abbreviations: ACC, advanced carbohydrate counting; BCC, basic carbohydrate counting; E%, percentage of total energy intake; HDL, high-density lipoprotein; Standard, standard care.
