# Supplementary material for: Comparing the Effectiveness of Different Dietary Educational Approaches for Carbohydrate Counting on Glycemic Control in Adults with Type 1 Diabetes: Findings from the DIET-CARB Study, a Randomized Controlled Trial
Source: Nutrients. 2024 Oct 31;16(21):3745. doi: 10.3390/nu16213745 (PMC11547945; doi:10.3390/nu16213745)
Supplement: Supplementary file 1 [file nutrients-16-03745-s001.zip › Table S3. Changes in GLP-1, antihypertensive, and lipid-lowering drugs during the study period.pdf]

Table S3 Participants prescribed GLP-1Ras, antihypertensive, and lipid-lowering medication at baseline, end-of-treatment, and follow-up

	**BCC** (*n = 18*)			**ACC** (*n = 18*)			**STANDARD** (*n = 17*)		
	Baseline	End-of-treatment	Follow-up	Baseline	End-of-treatment	Follow-up	Baseline	End-of-treatment	Follow-up
GLP-1Ras, n	1	1	1	1	0	0	1	0	0
Antihypertensive medication, n	5	6[1]	5[1]	3	3	3[3]	4	3[4]	4[4]
Lipid-lowering medication, n	5	4[2]	4[2]	8	8	8[3]	4	2[5]	2[5]

Abbreviations: ACC, advanced carbohydrate counting; BCC, basic carbohydrate counting; GLP-1RAs, glucagon-like peptide 1 receptor agonists.

[1]N = 1 initiated use during the intervention period and N=1 discontinued use during follow-up.

[2]N = 1 discontinued use in the intervention period, and N=1 discontinued and N=1 initiated use during follow-up.

[3]N = 1 discontinued use and N=1 initiated use during follow-up.

[4]N = 2 discontinued and N=1 initiated use during the intervention period, while N=1 discontinued and N=2 initiated use during follow-up.

[5]N = 2 discontinues use in the intervention period.
