# Supplementary material for: Comparing the Effectiveness of Different Dietary Educational Approaches for Carbohydrate Counting on Glycemic Control in Adults with Type 1 Diabetes: Findings from the DIET-CARB Study, a Randomized Controlled Trial
Source: Nutrients. 2024 Oct 31;16(21):3745. doi: 10.3390/nu16213745 (PMC11547945; doi:10.3390/nu16213745)
Supplement: Supplementary file 1 [file nutrients-16-03745-s001.zip › Table S4. Mealtime insulin adjustments at baseline, end-of-treatment and follow-up.pdf]

Table S4 Mealtime insulin adjustments at baseline, end-of-treatment and at follow-up

	BCC			ACC			STANDARD		
	Baseline *(N = 18)*	End-of treatment *(N = 14)*	Follow-up *(N = 15)*	Baseline *(N = 18)*	End-of treatment *(N = 15)*	Follow-up *(N = 14)*	Baseline *(N = 17)*	End-of treatment *(N = 15)*	Follow-up *(N = 13)*
**Breakfast**									
How often do you adjust your insulin dose for breakfast? (VAS 0-100)	72 (12,91)	89 (74,99)	71 (50,99)	82 (42,99)	87 (45,99)	73 (42,99)	75 (24,99)	90 (56,100)	81 (50,100)
Based on what method do you adjust your insulin dose at breakfast?									
- Calculation rules (carb count), n (%)	-	7 (50)	7 (47)	1 (6)	11 (73)	11 (79)	1 (6)	2 (13)	2
- Experience-based, n (%)	17 (94)	7 (50)	8 (53)	16 (89)	4 (27)	3 (21)	15 (88)	13 (87)	11
- Prescribed dose, n (%)	-	-	-	1 (6)	-	-	-	-	-
- Fixed insulin dose/same meals, n (%	1 (6)	-	-	-	-	-	1 (6)	-	-
**Lunch**									
How often do you adjust your insulin dose for lunch? (VAS 0-100)	75 (50,91)	86 (69, 99)	61 (50, 90)	79 (47, 99)	71 (52,90)	72 (57,83)	70 (50,99)	90 (70,100)	100 (65,100)
Based on what method do you adjust your insulin dose at lunch?									
- Calculation rules (carb count, n (%)	-	8 (57)	7 (47)	1 (6)	9 (60)	9 (60)	2 (12)	1 (7)	1 (8)
- Experience-based, n (%)	17 (94)	6 (43)	8 (53)	16 (89)	5 (33)	5 (40)	14 (82)	14 (93)	12 (92)
- Fixed insulin dose, n (%)	1 (6)	-	-	1 (6)	1 (7)	-	1 (6)	-	-
**Dinner**									
How often do you adjust your insulin dose for dinner? (VAS 0-100)	79 (50, 91)	94 (82,99)	76 (65,100)	84 (72, 100)	79 (67,97)	83 (63,95)	77 (63,99)	97 (74,100)	78 (73,100)
Based on what method do you adjust your insulin dose at dinner?									
- Calculation rules (carb count), n (%)	-	9 (64)	8 (53)	18 (100)	11 (73)	10 (71)	2 (12)	3 (20)	1 (8)
- Experience-based, n (%)	18 (100)	5 (36)	7 (47)	-	4 (27)	4 (29)	14 (82)	12 (80)	12 (92)
- Fixed dose, n (%)	-	-	-	-	-	-	1 (6)	-	-
**Between meals snack**									
How often do you adjust your insulin dose when snacking? (VAS 0-100)	50 (19,75)	77 (58 91)	77 (18,100)	57 (10, 91)	70 (16,89)	50 (50,83)	50 (4,73)	50 (20,90)	68 (12,92)
Based on what method do you adjust your insulin dose for snacks?									
- Calculation rules (carb count), n (%)	-	7 (50)	4 (27)	-	8 (53)	4 (29)	2 (12)	3 (20)	1 (8)
- Experience-based, n (%)	16 (88)	4 (29)	9 (60)	15 (83)	5 (33)	8 (57)	13 (76)	10 (67)	10 (77)
- Fixed dose, n (%)	-	-	-	-	-	-	1 (6)	-	-
- Never dose insulin for snacks, n (%)	2 (12)	3 (21)	2 (13)	3 (17)	2 (13)	2 (14)	1 (6)	2 (13)	2 (16)

VAS score are medians (25th and 75th percentiles). Categorical data are summarized by numbers and percentages. Abbreviations: ACC, advanced carbohydrate counting; BCC, basic carbohydrate counting; Standard, standard dietary care; VAS, Visual Analogue Scale.
