# Supplementary material for: Comparing the Effectiveness of Different Dietary Educational Approaches for Carbohydrate Counting on Glycemic Control in Adults with Type 1 Diabetes: Findings from the DIET-CARB Study, a Randomized Controlled Trial
Source: Nutrients. 2024 Oct 31;16(21):3745. doi: 10.3390/nu16213745 (PMC11547945; doi:10.3390/nu16213745)
Supplement: Supplementary file 1 [file nutrients-16-03745-s001.zip › Table S5. Baseline-adjusted estimates for primary and secondaryexploratory outcomes ¿C supplementary.pdf]

Table S5 Baseline-adjusted estimates for primary and secondary/exploratory outcomes – supplementary

Outcome	Group	Visit	Estimated mean (95% CI)	Within-group changes (95% CI)	Difference from Control (95% CI)	P-value
TBR 1, %*	ALL	Baseline	2.3 (1.6: 3.4)			
	Standard	EOT	2.6 (1.2: 5.6)	12.4 (-48.5: 145.0)		
	BCC	EOT	2.1 (0.9: 4.5)	-12.2 (-61.1: 98.4)	-21.8 (-73.5: 130.9)	0.646
	ACC	EOT	2.1 (1.0: 4.3)	-9.7 (-57.3: 91.0)	-19.6 (-71.4: 126.3)	0.670
TBR 2, %*	ALL	Baseline	1.7 (1.1: 2.8)			
	Standard	EOT	4.8 (1.9: 12.1)	175.1 (-3.6: 685.5)		
	BCC	EOT	2.5 (0.8: 7.5)	44.3 (-56.5: 379.3)	-47.5 (-87.8: 125.0)	0.363
	ACC	EOT	2.7 (1.0: 7.2)	53.6 (-49.0: 362.8)	-44.2 (-85.9: 121.9)	0.386
TAR 1, %	ALL	Baseline	25.1 (22.4: 27.8)			
	Standard	EOT	25.6 (19.6: 31.5)	0.5 (-5.6: 6.5)		
	BCC	EOT	26.0 (19.8: 32.2)	0.9 (-5.4: 7.2)	0.5 (-8.1: 9.0)	0.912
	ACC	EOT	24.0 (18.0: 29.9)	-1.1 (-7.2: 5.0)	-1.6 (-10.0: 6.8)	0.703
TAR 2, %	ALL	Baseline	13.3 (10.3: 16.2)			
	Standard	EOT	12.7 (7.5: 17.8)	-0.6 (-5.4: 4.2)		
	BCC	EOT	13.4 (8.1: 18.7)	0.1 (-4.9: 5.1)	0.7 (-6.2: 7.6)	0.836
	ACC	EOT	14.6 (9.4: 19.8)	1.3 (-3.6: 6.2)	1.9 (-4.9: 8.7)	0.576
SD, mmol/l	ALL	Baseline	3.7 (3.4: 4.0)			
	Standard	EOT	3.5 (3.0: 3.9)	-0.2 (-0.7: 0.2)		
	BCC	EOT	3.5 (3.1: 4.0)	-0.1 (-0.6: 0.3)	0.1 (-0.5: 0.7)	0.805
	ACC	EOT	3.6 (3.1: 4.0)	-0.1 (-0.6: 0.3)	0.1 (-0.5: 0.7)	0.728
CV, %	ALL	Baseline	39.5 (37.1: 41.9)			
	Standard	EOT	37.4 (32.4: 42.4)	-2.1 (-7.1: 2.8)		
	BCC	EOT	38.4 (33.2: 43.5)	-1.1 (-6.3: 4.0)	1.0 (-6.1: 8.0)	0.778
	ACC	EOT	39.1 (34.1: 44.1)	-0.4 (-5.4: 4.6)	1.7 (-5.2: 8.6)	0.619
BMI, kg/m2	ALL	Baseline	26.5 (25.6: 27.3)			
	Standard	EOT	26.5 (25.6: 27.5)	0.1 (-0.4: 0.5)		
		Follow-up	26.3 (25.3: 27.3)	-0.2 (-0.8: 0.4)		
	BCC	EOT	26.6 (25.7: 27.5)	0.1 (-0.3: 0.6)	0.1 (-0.6: 0.8)	0.820
		Follow-up	26.8 (25.9: 27.8)	0.4 (-0.2: 1.0)	0.6 (-0.3: 1.4)	0.174
	ACC	EOT	26.5 (25.5: 27.4)	-0.0 (-0.5: 0.5)	-0.1 (-0.7: 0.6)	0.836
		Follow-up	26.8 (25.8: 27.8)	0.3 (-0.3: 0.9)	0.5 (-0.4: 1.4)	0.253
Body weight, kg	ALL	Baseline	81.8 (78.3: 85.3)			
	Standard	EOT	81.7 (78.2: 85.3)	-0.1 (-1.5: 1.4)		
		Follow-up	81.1 (77.3: 84.8)	-0.7 (-2.6: 1.2)		
	BCC	EOT	82.4 (78.9: 85.9)	0.6 (-0.9: 2.1)	0.6 (-1.4: 2.7)	0.539
		Follow-up	83.0 (79.3: 86.8)	1.2 (-0.6: 3.1)	2.0 (-0.7: 4.6)	0.140
	ACC	EOT	81.7 (78.1: 85.2)	-0.1 (-1.6: 1.3)	-0.1 (-2.2: 2.0)	0.935

Outcome	Group	Visit	Estimated mean (95% CI)	Within-group changes (95% CI)	Difference from Control (95% CI)	P-value
		Follow-up	82.7 (79.0: 86.5)	0.9 (-1.0: 2.8)	1.7 (-1.0: 4.3)	0.222
Average waist, cm	ALL	Baseline	92 (89: 95)			
	Standard	EOT	92 (88: 96)	-0 (-4: 3)		
		Follow-up	91 (87: 95)	-1 (-5: 2)		
	BCC	EOT	94 (90: 98)	2 (-1: 5)	2 (-2: 7)	0.286
		Follow-up	95 (91: 99)	3 (-1: 6)	4 (-1: 9)	0.114
	ACC	EOT	92 (88: 96)	-0 (-4: 3)	0 (-4: 5)	0.966
		Follow-up	93 (88: 97)	0 (-3: 4)	2 (-3: 7)	0.473
WHR, unitless	ALL	Baseline	0.99 (0.96: 1.03)			
	Standard	EOT	0.98 (0.94: 1.01)	-0.02 (-0.05: 0.01)		
		Follow-up	0.98 (0.95: 1.02)	-0.01 (-0.04: 0.02)		
	BCC	EOT	0.97 (0.93: 1.01)	-0.02 (-0.05: 0.01)	-0.01 (-0.05: 0.04)	0.788
		Follow-up	0.96 (0.93: 1.00)	-0.03 (-0.06: 0.00)	-0.02 (-0.06: 0.02)	0.306
	ACC	EOT	0.99 (0.96: 1.03)	0.00 (-0.03: 0.03)	0.02 (-0.02: 0.06)	0.357
		Follow-up	0.99 (0.95: 1.02)	-0.00 (-0.03: 0.03)	0.00 (-0.03: 0.04)	0.823
SBP, mmHg	ALL	Baseline	125 (122: 128)			
	Standard	EOT	125 (120: 130)	-1 (-5: 4)		
		Follow-up	125 (119: 130)	-1 (-6: 5)		
	BCC	EOT	127 (122: 133)	2 (-3: 7)	2 (-4: 9)	0.472
		Follow-up	125 (120: 131)	-0 (-5: 5)	1 (-7: 8)	0.870
	ACC	EOT	123 (118: 129)	-2 (-7: 3)	-1 (-8: 5)	0.693
		Follow-up	124 (118: 129)	-2 (-7: 3)	-1 (-8: 6)	0.793
DBP, mmHg	ALL	Baseline	77 (75: 80)			
	Standard	EOT	75 (72: 79)	-2 (-4: 1)		
		Follow-up	77 (74: 80)	0 (-3: 3)		
	BCC	EOT	78 (75: 82)	1 (-2: 4)	3 (-1: 7)	0.172
		Follow-up	77 (74: 80)	0 (-2: 3)	0 (-3: 4)	0.924
	ACC	EOT	77 (74: 81)	0 (-2: 3)	2 (-2: 6)	0.311
		Follow-up	80 (77: 83)	3 (0: 5)	3 (-1: 6)	0.156
TC, mmol/l	ALL	Baseline	4.4 (4.2: 4.6)			
	Standard	EOT	4.3 (4.0: 4.6)	-0.1 (-0.4: 0.1)		
		Follow-up	4.4 (4.0: 4.8)	0.0 (-0.4: 0.4)		
	BCC	EOT	4.5 (4.2: 4.8)	0.1 (-0.1: 0.4)	0.3 (-0.1: 0.6)	0.153
		Follow-up	4.2 (3.8: 4.5)	-0.2 (-0.6: 0.1)	-0.2 (-0.8: 0.3)	0.370
	ACC	EOT	4.2 (4.0: 4.5)	-0.2 (-0.4: 0.1)	-0.0 (-0.4: 0.4)	0.962
		Follow-up	4.3 (4.0: 4.7)	-0.1 (-0.4: 0.3)	-0.1 (-0.6: 0.5)	0.785
LDL-C, mmol/l	ALL	Baseline	2.4 (2.2: 2.6)			
	Standard	EOT	2.4 (2.1: 2.6)	-0.1 (-0.3: 0.2)		

Outcome	Group	Visit	Estimated mean (95% CI)	Within-group changes (95% CI)	Difference from Control (95% CI)	P-value
		Follow-up	2.4 (2.1: 2.7)	-0.0 (-0.3: 0.3)		
	BCC	EOT	2.6 (2.3: 2.8)	0.1 (-0.1: 0.4)	0.2 (-0.1: 0.5)	0.227
		Follow-up	2.4 (2.1: 2.7)	-0.0 (-0.3: 0.3)	0.0 (-0.4: 0.4)	0.973
	ACC	EOT	2.3 (2.0: 2.5)	-0.2 (-0.4: 0.1)	-0.1 (-0.4: 0.2)	0.581
		Follow-up	2.4 (2.1: 2.7)	-0.0 (-0.3: 0.3)	0.0 (-0.4: 0.4)	0.911
HDL-C, mmol/l	ALL	Baseline	1.53 (1.41: 1.65)			
	Standard	EOT	1.46 (1.31: 1.60)	-0.08 (-0.19: 0.03)		
		Follow-up	1.53 (1.38: 1.68)	-0.00 (-0.11: 0.11)		
	BCC	EOT	1.54 (1.39: 1.69)	0.01 (-0.10: 0.12)	0.09 (-0.07: 0.24)	0.279
		Follow-up	1.54 (1.39: 1.68)	0.00 (-0.10: 0.11)	0.01 (-0.15: 0.16)	0.940
	ACC	EOT	1.63 (1.48: 1.78)	0.10 (-0.01: 0.21)	0.17 (0.02: 0.33)	0.029
		Follow-up	1.58 (1.43: 1.72)	0.05 (-0.06: 0.15)	0.05 (-0.11: 0.20)	0.530
Triglyceride, mmol/l*	ALL	Baseline	0.87 (0.77: 0.98)			
	Standard	EOT	0.87 (0.72: 1.05)	0.07 (-15.47: 18.46)		
		Follow-up	0.94 (0.78: 1.13)	8.55 (-7.74: 27.72)		
	BCC	EOT	0.91 (0.76: 1.10)	4.92 (-11.46: 24.33)	4.85 (-17.25: 32.86)	0.689
		Follow-up	0.88 (0.74: 1.05)	1.69 (-12.82: 18.63)	-6.32 (-25.04: 17.08)	0.558
	ACC	EOT	0.82 (0.68: 0.98)	-5.99 (-20.59: 11.29)	-6.05 (-25.80: 18.95)	0.596
		Follow-up	-0.22 (-0.40: -0.04)	-0.08 (-0.23: 0.08)	-0.16 (-0.38: 0.07)	0.159
Total energy, kJ/day	ALL	Baseline	8297 (7651: 8942)			
	Standard	EOT	8686 (7817: 9556)	390 (-432: 1211)		
	BCC	EOT	8224 (7380: 9068)	-73 (-867: 721)	-462 (-1557: 633)	0.398
	ACC	EOT	6483 (5662: 7304)	-1814 (-2583: -1044)	-2204 (-3281: -1126)	0.000
Carbohydrates, E%	ALL	Baseline	43 (41: 45)			
	Standard	EOT	46 (42: 49)	3 (-1: 6)		
	BCC	EOT	44 (41: 48)	1 (-3: 5)	-2 (-7: 4)	0.539
	ACC	EOT	44 (41: 48)	1 (-2: 5)	-1 (-6: 4)	0.593
Carbohydrates, g/day	ALL	Baseline	195 (180: 211)			
	Standard	EOT	211 (186: 236)	16 (-7: 39)		
	BCC	EOT	203 (179: 228)	8 (-14: 30)	-8 (-39: 23)	0.606
	ACC	EOT	166 (143: 190)	-29 (-50: -8)	-45 (-76: -15)	0.005
Fat, E%	ALL	Baseline	35 (33: 37)			
	Standard	EOT	34 (31: 37)	-1 (-4: 2)		
	BCC	EOT	36 (33: 38)	0 (-3: 3)	1 (-2: 5)	0.473
	ACC	EOT	32 (30: 35)	-3 (-6: -0)	-2 (-5: 2)	0.350
Protein, E%	ALL	Baseline	19 (18: 19)			
	Standard	EOT	16 (14: 18)	-2 (-4: -0)		
	BCC	EOT	17 (15: 19)	-2 (-3: 0)	1 (-2: 3)	0.652

Outcome	Group	Visit	Estimated mean (95% CI)	Within-group changes (95% CI)	Difference from Control (95% CI)	P-value
	ACC	EOT	20 (18: 22)	2 (-0: 3)	4 (1: 6)	0.005
SFA, g/day	ALL	Baseline	28 (25: 31)			
	Standard	EOT	30 (26: 34)	2 (-2: 7)		
	BCC	EOT	27 (23: 30)	-1 (-5: 3)	-4 (-9: 2)	0.160
	ACC	EOT	20 (16: 24)	-8 (-12: -4)	-10 (-16: -5)	0.000
MUFA, g/day*	ALL	Baseline	21 (18: 24)			
	Standard	EOT	21 (18: 26)	3 (-16: 26)		
	BCC	EOT	20 (16: 24)	-5 (-21: 16)	-7 (-28: 19)	0.543
	ACC	EOT	15 (12: 18)	-28 (-40: -13)	-30 (-46: -10)	0.006
PUFA, g/day	ALL	Baseline	10 (9: 12)			
	Standard	EOT	11 (9: 13)	1 (-2: 3)		
	BCC	EOT	10 (8: 12)	-1 (-3: 2)	-1 (-4: 1)	0.320
	ACC	EOT	7 (6: 9)	-3 (-5: -1)	-4 (-6: -1)	0.009
Dietary fiber, g/day*	ALL	Baseline	23 (20: 25)			
	Standard	EOT	24 (20: 28)	5 (-12: 26)		
	BCC	EOT	20 (17: 24)	-11 (-25: 6)	-15 (-33: 7)	0.160
	ACC	EOT	18 (15: 21)	-20 (-32: -5)	-23 (-39: -4)	0.023
Dietary fiber, g/10 MJ*	ALL	Baseline	28 (26: 31)			
	Standard	EOT	27 (23: 33)	-3 (-19: 16)		
	BCC	EOT	26 (22: 31)	-9 (-24: 9)	-6 (-26: 20)	0.614
	ACC	EOT	29 (24: 34)	3 (-14: 22)	6 (-17: 35)	0.635
Added sugar, g/day	ALL	Baseline	53 (46: 60)			
	Standard	EOT	56 (44: 69)	3 (-10: 15)		
	BCC	EOT	61 (49: 73)	7 (-5: 19)	5 (-12: 21)	0.588
	ACC	EOT	42 (30: 54)	-11 (-23: 0)	-14 (-31: 2)	0.092
Added sugar, g/10 MJ	ALL	Baseline	64 (57: 72)			
	Standard	EOT	67 (51: 84)	3 (-14: 20)		
	BCC	EOT	74 (59: 90)	10 (-6: 26)	7 (-16: 30)	0.526
	ACC	EOT	62 (47: 77)	-2 (-18: 14)	-5 (-27: 17)	0.651
MET minutes/week*	ALL	Baseline	2258 (1631: 3127)			
	Standard	EOT	2819 (1795: 4427)	25 (-21: 97)		
		Follow-up	2598 (1521: 4440)	15 (-34: 99)		
	BCC	EOT	2274 (1446: 3575)	1 (-36: 58)	-19 (-56: 48)	0.474
		Follow-up	2133 (1254: 3628)	-6 (-45: 61)	-18 (-61: 71)	0.589
	ACC	EOT	1627 (1045: 2533)	-28 (-54: 12)	-42 (-68: 5)	0.070
		Follow-up	1692 (1007: 2843)	-25 (-56: 26)	-35 (-68: 34)	0.238

Estimated means (CI 95%) (left) and baseline corrected difference between groups (CI 95%). P-values are between group differences. *Variable has been log-transformed for analysis and back−transformed for presentation. **Abbreviations:** ACC, advanced carbohydrate counting; BCC basic carbohydrate counting, BMI, body mass index; CV, coefficient of variation; DBP, Diastolic blood pressure; EOT, End-of-treatment after six months of intervention; HDL-C, high density lipoprotein cholesterol, LDL-C, low-density lipoprotein

cholesterol, MET, Metabolic Equivalent of Task; MUFA, monounsaturated fatty acids; PUFA, polyunsaturated fatty acids; SBP, Systolic blood pressure; SD, standard deviation; SFA, saturated fatty acids; Standard, standard dietary treatment; TAR 1, time above range at level 1 (10.0-13.9 mmol/l) % spent of time; TAR 2, time above range at level 2 (>13.9 mmol/l) % spent of time; TBR 1, time below range at level 1 (3.9-3.0 mmol/l) % spent of time; TBR 2, time below range at level 2 (< 3 mmol/l) % spent of time; TC, total cholesterol; WHR, Waist/Hip ratio.
