# Supplementary material for: Comparing the Effectiveness of Different Dietary Educational Approaches for Carbohydrate Counting on Glycemic Control in Adults with Type 1 Diabetes: Findings from the DIET-CARB Study, a Randomized Controlled Trial
Source: Nutrients. 2024 Oct 31;16(21):3745. doi: 10.3390/nu16213745 (PMC11547945; doi:10.3390/nu16213745)
Supplement: Supplementary file 1 [file nutrients-16-03745-s001.zip › Table S6. Delta values for DDQOL, PCDS, HCCQ.pdf]

Table S6 Delta values for diabetes diet-related quality of life, perceived autonomy support and competencies in diet and diabetes

Outcomes	Group	Visit	Median (IQR)	P-value
**DDQOL subscales**				
Dietary satisfaction	Standard	End-of-treatment	0.0 (0.0; 9.38)	
	BCC		0.0 (-4.7; 12.5)	0.753
	ACC		0.0 (0.0; 9.4)	0.569
Dietary benefits	Standard	End-of-treatment	0.0 (-5.0; 12.5)	
	BCC		5.0 (-5.0; 5.0)	0.807
	ACC		5.0 (-2.5; 7.5)	0.899
Dietary burden	Standard	End-of-treatment	3.1 (0.0; 10.9)	
	BCC		4.7 (-3.1; 11.7)	0.860
	ACC		3.1 (-3.1; 12.5)	0.786
Social restrictions	Standard	End-of-treatment	0.0 (0.0; 0.0)	
	BCC		0.0 (-25.0; 0.0)	0.176
	ACC		0.0 (-12.5; 0.0)	0.753
General perception of diet	Standard	End-of-treatment	25.0 (0.0; 25.0)	
	BCC		0.0 (0.0; 0.0)	0.017
	ACC		0.0 (0.0; 12.5)	0.153
Mental health	Standard	End-of-treatment	10.0 (5.0;12.5)	
	BCC		-2.5 (-5.0; 3.8)	0.019
	ACC		0.0 (-5.0; 7.5)	0.069
Vitality	Standard	End-of-treatment	12.5 (6.3; 18.8)	
	BCC		0.0 (0.0; 4.7)	0.014
	ACC		-6.3 (-15.6; 9.4)	0.035
**PCDS**				
	Standard	End-of-treatment	5.0 (1.5; 7.0)	
	BCC		6.0 (0.3; 8.0)	0.861
	ACC		5.0 (2.5; 9.5)	0.983
**HCCQ**	Standard	End-of-treatment	0.6 (0.4; 1.6)	
	BCC		1.1 (0.4; 2.7)	0.326
	ACC		1.2 (0.0; 1.8)	0.865

Delta values (median and IQR 25th to 75th percentiles) for changes in summed scores from baseline to end-of-treatment between groups. P-values are between group differences.
Abbreviations: ACC, advanced carbohydrate counting; BCC, basic carbohydrate counting; DDQOL, diabetes diet-related quality of life; HCCQ, health-care climate questionnaire in relation to dietitian support; PCDS, Perceived competencies in diet and diabetes; Standard, standard dietary treatment.
