# Supplementary figures and images for: Comparing the Effectiveness of Different Dietary Educational Approaches for Carbohydrate Counting on Glycemic Control in Adults with Type 1 Diabetes: Findings from the DIET-CARB Study, a Randomized Controlled Trial
Source: Nutrients. 2024 Oct 31;16(21):3745. doi: 10.3390/nu16213745 (PMC11547945; doi:10.3390/nu16213745)

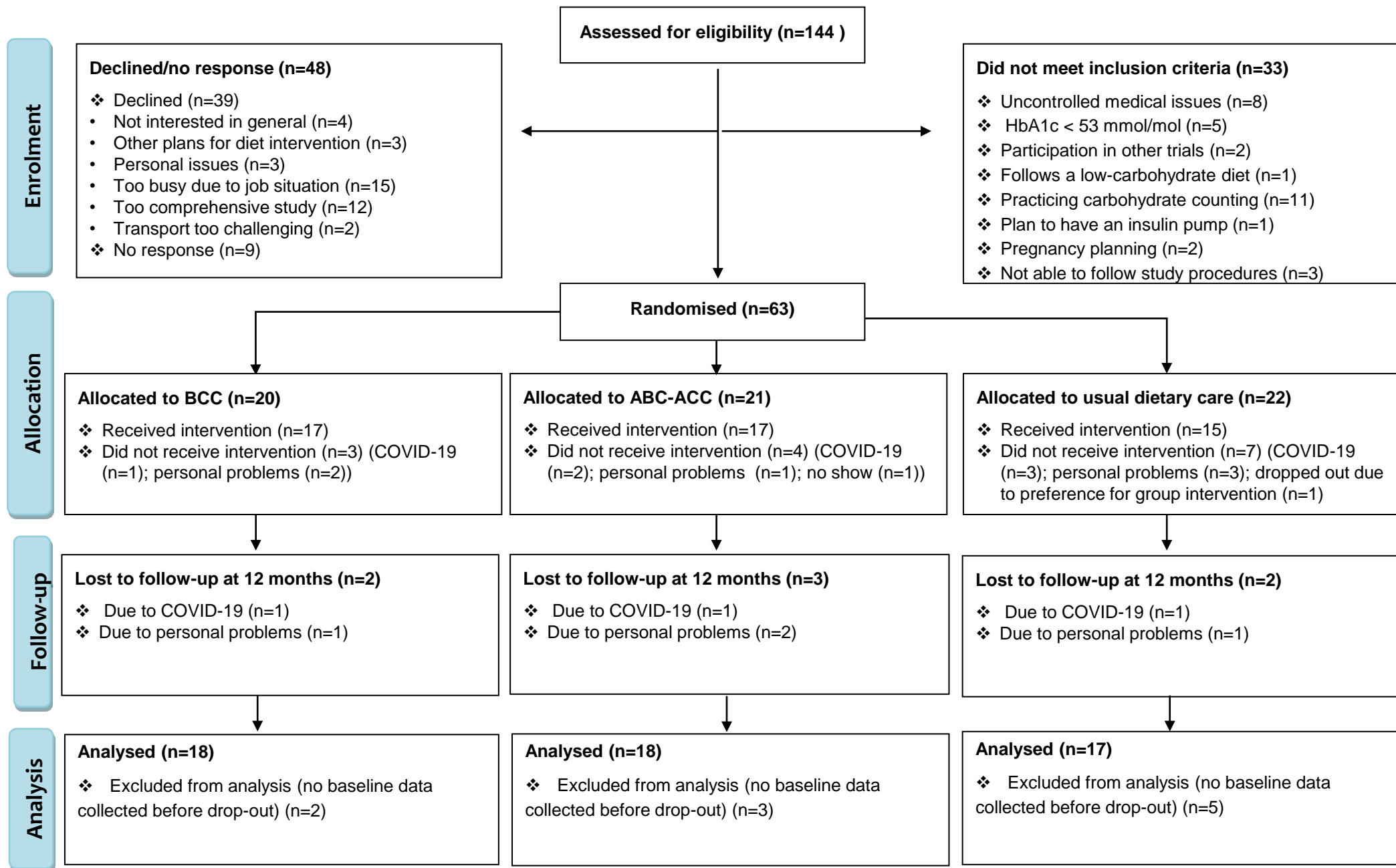

**Fig. S1** Flow diagram DIET-CARB Study

Supplement: Supplementary file 1 [file nutrients-16-03745-s001.zip › Figure S1 Flow diagram.pdf]
